# Risk factors associated with severe hospital burden of COVID-19 disease in Regione Lombardia: a cohort study

**DOI:** 10.1186/s12879-021-06750-z

**Published:** 2021-10-07

**Authors:** Anne M. Presanis, Kevin Kunzmann, Francesca M. Grosso, Christopher H. Jackson, Alice Corbella, Giacomo Grasselli, Marco Salmoiraghi, Maria Gramegna, Daniela De Angelis, Danilo Cereda

**Affiliations:** 1 Medical Research Council Biostatistics Unit, School of Clinical Medicine, University of Cambridge, Cambridge, UK; 2 Postgraduate School of Public Health, Department of Biomedical Sciences for Health, University of Milan, Milan, Italy; 3 University of Warwick, Coventry, UK; 4 Fondazione IRCCS Ca’ Granda Ospedale Maggiore Policlinico, University of Milan, Milan, Italy; 5 Welfare General Directorate, Regione Lombardia, Milan, Italy

**Keywords:** COVID-19, Hospital-fatality risk, Critical care, Multi-state model, Mixture model

## Abstract

**Background:**

Understanding the risk factors associated with hospital burden of COVID-19 is crucial for healthcare planning for any future waves of infection.

**Methods:**

An observational cohort study is performed, using data on all PCR-confirmed cases of COVID-19 in Regione Lombardia, Italy, during the first wave of infection from February-June 2020. A multi-state modelling approach is used to simultaneously estimate risks of progression through hospital to final outcomes of either death or discharge, by pathway (via critical care or not) and the times to final events (lengths of stay). Logistic and time-to-event regressions are used to quantify the association of patient and population characteristics with the risks of hospital outcomes and lengths of stay respectively.

**Results:**

Risks of severe outcomes such as ICU admission and mortality have decreased with month of admission (for example, the odds ratio of ICU admission in June vs March is 0.247 [0.120–0.508]) and increased with age (odds ratio of ICU admission in 45–65 vs 65 + age group is 0.286 [0.201–0.406]). Care home residents aged 65 + are associated with increased risk of hospital mortality and decreased risk of ICU admission. Being a healthcare worker appears to have a protective association with mortality risk (odds ratio of ICU mortality is 0.254 [0.143–0.453] relative to non-healthcare workers) and length of stay. Lengths of stay decrease with month of admission for survivors, but do not appear to vary with month for non-survivors.

**Conclusions:**

Improvements in clinical knowledge, treatment, patient and hospital management and public health surveillance, together with the waning of the first wave after the first lockdown, are hypothesised to have contributed to the reduced risks and lengths of stay over time.

**Supplementary Information:**

The online version contains supplementary material available at 10.1186/s12879-021-06750-z.

## Background

Since the start of the coronavirus disease 2019 (COVID-19) pandemic, understanding the severe burden on healthcare, particularly hospitals, and the risk factors associated with severe events has been a crucial question. Note that by “risk factors” we mean covariates significantly associated with the outcome. Initial research [1–4] demonstrates that older age (> 60), male sex and having pre-existing medical conditions (comorbidities) are risk factors associated with severe events in hospitalized COVID-19 patients, such as intensive care unit (ICU) admission, intubation and mortality. However, much of this early work used standard survival analysis, without accounting for the competing risks of different pathways through hospital (e.g. death without ICU vs ICU vs recovery without ICU; or death in ICU vs recovery & transfer to a post-ICU ward). Ignoring competing outcomes can bias estimates of risks such as the hospital-fatality risk—the probability that a hospital admission for COVID-19 disease can lead to death—and therefore of effect sizes [5]. While analyses accounting for competing events have been performed in other countries [6, 7], to our knowledge, only standard survival analysis has been used for data collected in Italy. We therefore present a comprehensive study of risk factors associated with progression through hospital to either discharge or death, via ICU or not, while accounting for competing risks.

The first Italian individual confirmed positive for the novel SARS-CoV-2 virus was identified on 20th February 2020 in Codogno, Regione Lombardia. Confirmed cases of COVID-19 rapidly increased to 403 in just one week [8], peaking on 20th March 2020 and causing a pressure build-up on the regional healthcare system. Efforts were directed to expanding the number of beds, especially in ICU [9]. Using a “hub-and-spoke” model [10], suspected COVID-19 patients were admitted to one of 17 designated hub hospitals in the Infectious Disease Hospital Network, chosen due to their skill in the fields of infectious disease and intensive care. In Lombardy a substantial proportion of patients were treated in hospital rather than at home [11] and treatment changed according to the severity of symptoms: no treatment was suggested for asymptomatic or mildly symptomatic patients with no comorbidities; therapy for symptoms and oxygen support were indicated for at-risk-of-progression patients with mild symptoms or with moderate symptoms; while critically ill patients were referred to ICU [12]. During the first wave, the highest number of patients admitted to hospital per day was reached in mid-March, with 838 patients, of whom 56 were admitted to ICU [13]. In addition to the “hub-and-spoke” model implemented, the Prevention Unit of the General Directorate of Welfare of Regione Lombardia coordinated data collection from laboratories and healthcare on all confirmed cases of COVID-19 into a single id-hinged register, the COVID-19 Regional Database [14].

The aims of this study are to assess: the magnitude of the burden of the COVID-19 pandemic on Lombardia secondary healthcare in terms of risks of severe outcomes and lengths of stay in hospital; and how this burden varies across demographic and health groups. Use of the subset of cases in the COVID-19 Regional Database who were hospitalised allows quantification of the within-hospital burden using a multi-state modelling approach [15] to account for competing risks. We simultaneously estimate the risks of each possible pathway through hospital (with or without ICU admission, to either discharge or death) and the times taken to reach each outcome (lengths of stay both overall and in different wards: pre-ICU, ICU and post-ICU). Detailed patient information allows assessment of the risk factors associated with progression through hospital and outcomes. Crucially, we assess not only patient characteristics such as age and sex, but also population-level risk factors: (i) month of admission, as a proxy for the external state and public health management of the epidemic and changes in clinical treatment and hospital management of cases; and (ii) hospital size, as a proxy for Regione Lombardia’s use of the “hub-and-spoke” model.

## Methods

### Study participants

The Lombardia COVID-19 Regional Database, as of 5th August 2020 and covering the period 1st December 2019 to 17th July 2020, contains detailed pseudo-anonymised retrospective individual-level data on the cohort of all individuals in Regione Lombardia diagnosed with COVID-19 via polymerase chain reaction (PCR) during the first wave. This dataset is described in full elsewhere [10, 14] (see also Additional file 1: Appendix S1), but briefly, records age, sex, Local Healthcare Agency district of Lombardia, presence of co-morbidities, whether the individual is a healthcare worker or care home resident, presence of symptoms, whether or not the individual was hospitalised, and if so, details of the admitting hospital. For each individual, dates of symptom onset, positive laboratory test, hospital admission, ICU admission, ICU discharge, hospital discharge, recovery and death are recorded. Here the subset of 40,550 patients who were admitted to hospital between February and June, with known admission date and admitting hospital, and who had consistent date information are analysed (Additional file 1: Appendix S1, Figure S1). Seven key covariates describing patient and hospital characteristics are considered to understand risk factors associated with progression through hospital. In order of hypothesised importance, these are: age group (0–45, 46–65, 66 +); sex (male, female); month of admission (Feb, Mar, Apr, May, Jun); presence of at least one co-morbidity (defined as a pre-existing medical condition and categorised as respiratory, cardio-vascular, metabolic or oncological, see Additional file 1: Appendix S1); care home resident (yes, no); healthcare worker (yes, no); and hospital bed capacity (large, medium or small, defined according to the numbers of hospital and ICU beds, see Additional file 1: Appendix S1, Table S1). All methods detailed below were performed in accordance with the relevant guidelines and regulations governing these data.

### Multi-state model

**Fig. 1:**
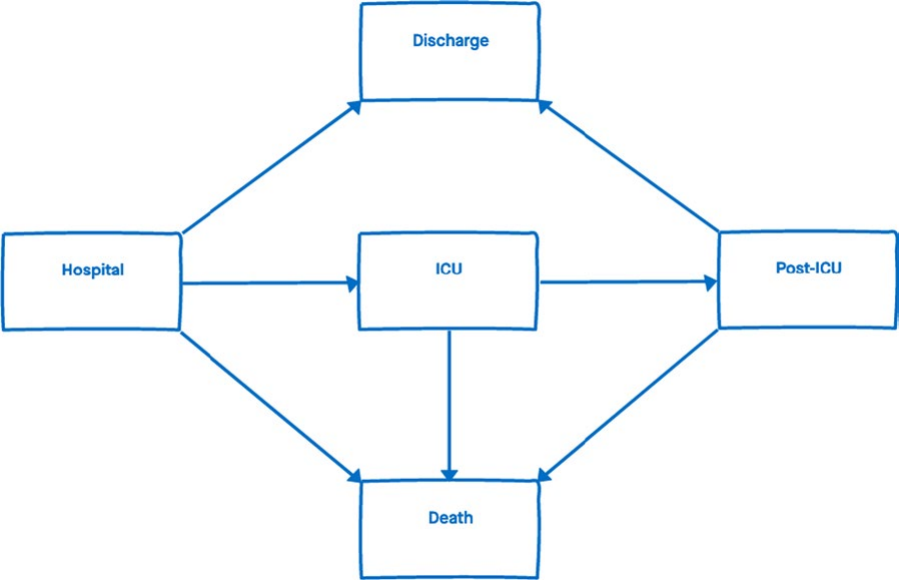
Multi-state model

As in Grosso, Presanis et al. (2021) [10], the progression of patients through hospital can be represented by a multi-state model with states Hospital, ICU, Post-ICU, Discharge, and Death (Fig. 1). The outcomes of interest are: the probabilities of entering each state (a “next event”) given the current state; the probabilities of final events, either Death (“*hospital-fatality risk*”, as defined in Additional file 1: Appendix S4) or Discharge, given hospital admission or the current state; and corresponding times to each next event, conditional on experiencing the next event, i.e. the lengths of stay (LoS) in each state; and the total LoS in hospital.

The type of multi-state model considered is a “mixture model” [15, 16], combining multinomial or binomial logistic regression of probabilities of different pathways through hospital on covariates with parametric time-to-event analyses for each LoS in a state, conditional on the next event occurring.

In the dataset considered, outcomes are missing for < 1% of individuals in the Hospital and ICU states, and for 15% of individuals in the Post-ICU state. It is unknown whether the missing outcomes had not happened by the end-date of the data, 17th July, ("right-censoring"), or whether the outcome had happened, but was not recorded ("missing data/loss to follow-up"). Grosso, Presanis et al. [10] considered two alternative assumptions for the Lombardia dataset, since it is impossible to distinguish between censoring and missing outcomes: (1) the missing outcomes are ignorable; (2) these individuals are right-censored at one day after their last observed event. The results are very similar under the two assumptions, so in what follows, the results under assumption (1) are reported for models with multiple covariates, since computation for multivariable models is more feasible under assumption (1). This computation is feasible because by ignoring missing outcomes, the multi-state model can be split into its component parts and implemented in a modular way: the logistic regression models for the probabilities; and the parametric time-to-event models.

The factorised models are fitted using a covariate selection procedure, as detailed in the following sections. Point estimates and 95% confidence intervals (CIs) are reported for each probability of interest, as well as for the median and interquartile ranges (IQRs) of each time-to-event distribution. The confidence intervals represent parameter uncertainty, whereas the median and IQRs summarise the heterogeneity across individuals in the time-to-event distributions.

All analyses were carried out in R version 3.6.3, using the *flexsurv* package [15].

### Probabilities of next events

Multinomial logistic regression is carried out for the transition probabilities from hospital admission to the next events (discharge, ICU admission, death without ICU admission). Binomial logistic regression is employed for the transition probabilities: from ICU to post-ICU or death; and from a post-ICU stay to discharge or death.

Multivariable models with main effects only are first considered, adding covariates one at a time, in order of hypothesised importance. The model that minimises Akaike’s Information Criterion (AIC) is selected. To investigate interactions, four additional models are considered, based on what interactions are expected to be important: (a) age group interacting with the covariates selected at the previous step; (b) month of admission interacting with the covariates selected at the previous step; (c) age group interacting with all other covariates; (d) month of admission interacting with all other covariates. The final model is chosen among the interim selected model without interactions and the interaction models (a)-(d) as the one that minimises the AIC.

### Times to next events

Conditional on patients experiencing each next event, parametric models are fit independently for each time from a hospital admission, ICU admission or post-ICU state to the next event (i.e. each LoS). A two-step procedure is used to select a model for each transition, where first covariates (as above), then parametric distributions (generalised gamma, gamma, log-normal or Weibull) are chosen according to AIC and likelihood ratio tests.

## Results

### Data summaries

Covariate summaries for the 40,550 patients in the Lombardia hospital cohort are shown in Table 1. Patients in this cohort had a median age of 69 (interquartile range [56–80]), with the youngest aged 0 and the oldest aged 102.

**Table 1:** Covariate summaries

Covariate	Level	Number	Proportion (%)
Age group	[0,45]	4236	10.45
	(45,65]	13,010	32.08
	(65,Inf]	23,304	57.47
Sex	Female	16,063	39.61
	Male	24,487	60.39
Month of admission	Feb	1606	3.96
	Mar	28,101	69.30
	Apr	8813	21.73
	May	1525	3.76
	Jun	505	1.25
At least 1 co-morbidity?	No	14,787	36.47
	Yes	25,763	63.53
Care home resident?	No	38,825	95.75
	Yes	1725	4.25
Healthcare worker?	No	38,889	95.90
	Yes	1661	4.10
Hospital bed capacity	Large	28,663	70.69
	Medium	5288	13.04
	Small	6599	16.27

**Fig. 2:**
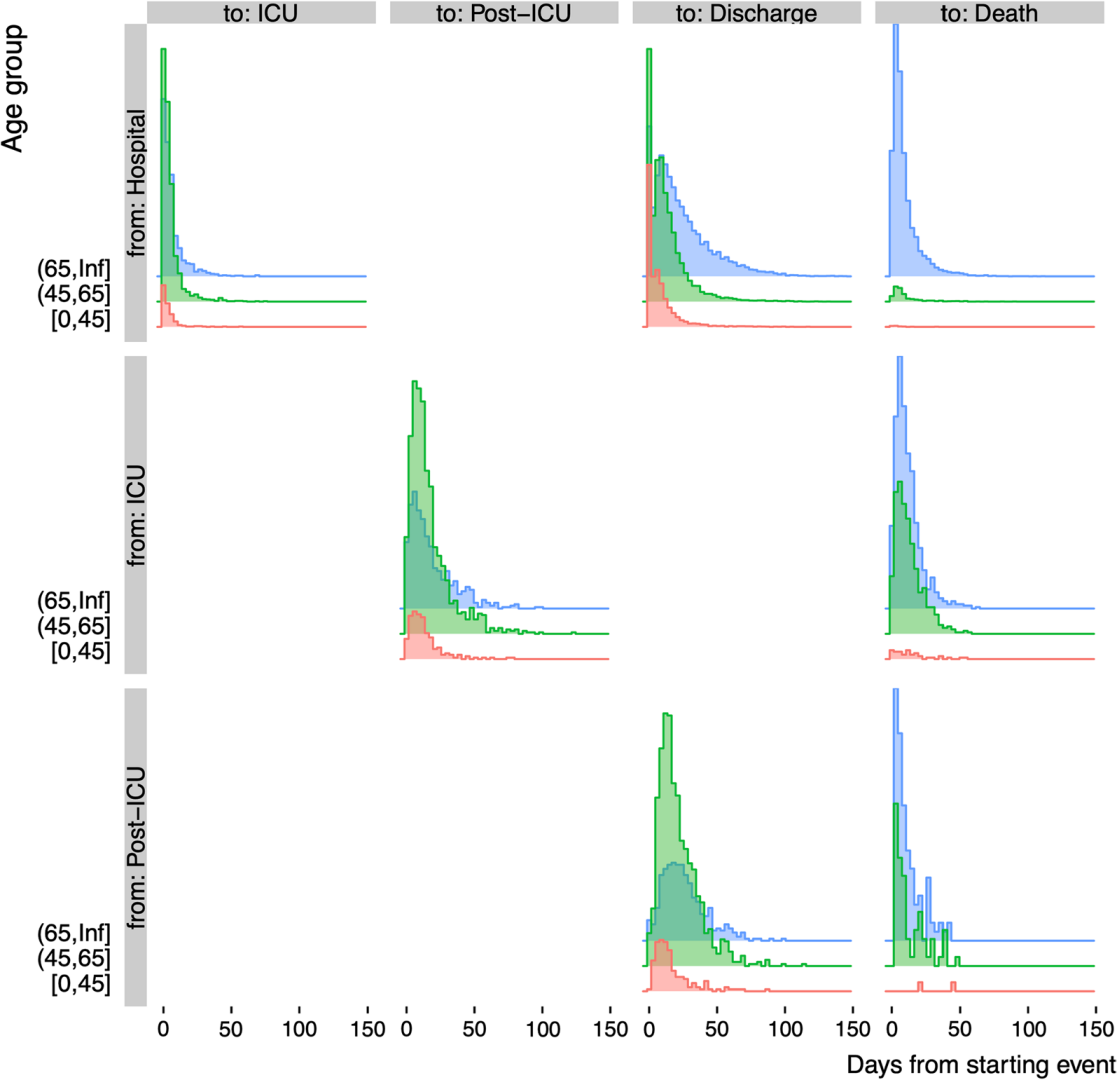
Observed distributions of lengths of stay: times from starting states (hospital admission, ICU admission, post-ICU stay: rows) to next events (ICU admission, a post-ICU stay, discharge and death: columns), by age group (colours, y-axis). Vertical scale proportional to number of individuals

Figure 2 displays, by age group, the empirical distributions of the times between events, namely LoS in the Hospital, ICU and Post-ICU states, by subsequent events.

### Month of admission trends by age and sex

The selected models for the transition probabilities and times-to-next-events (distributions and covariates selected) are summarised in Additional file 1: Tables S2 and S6. Covariate effects in terms of odds ratios (ORs) and expected time ratios (ETRs) are fully reported in Additional files 1: S2 and S3.

**Fig. 3:**
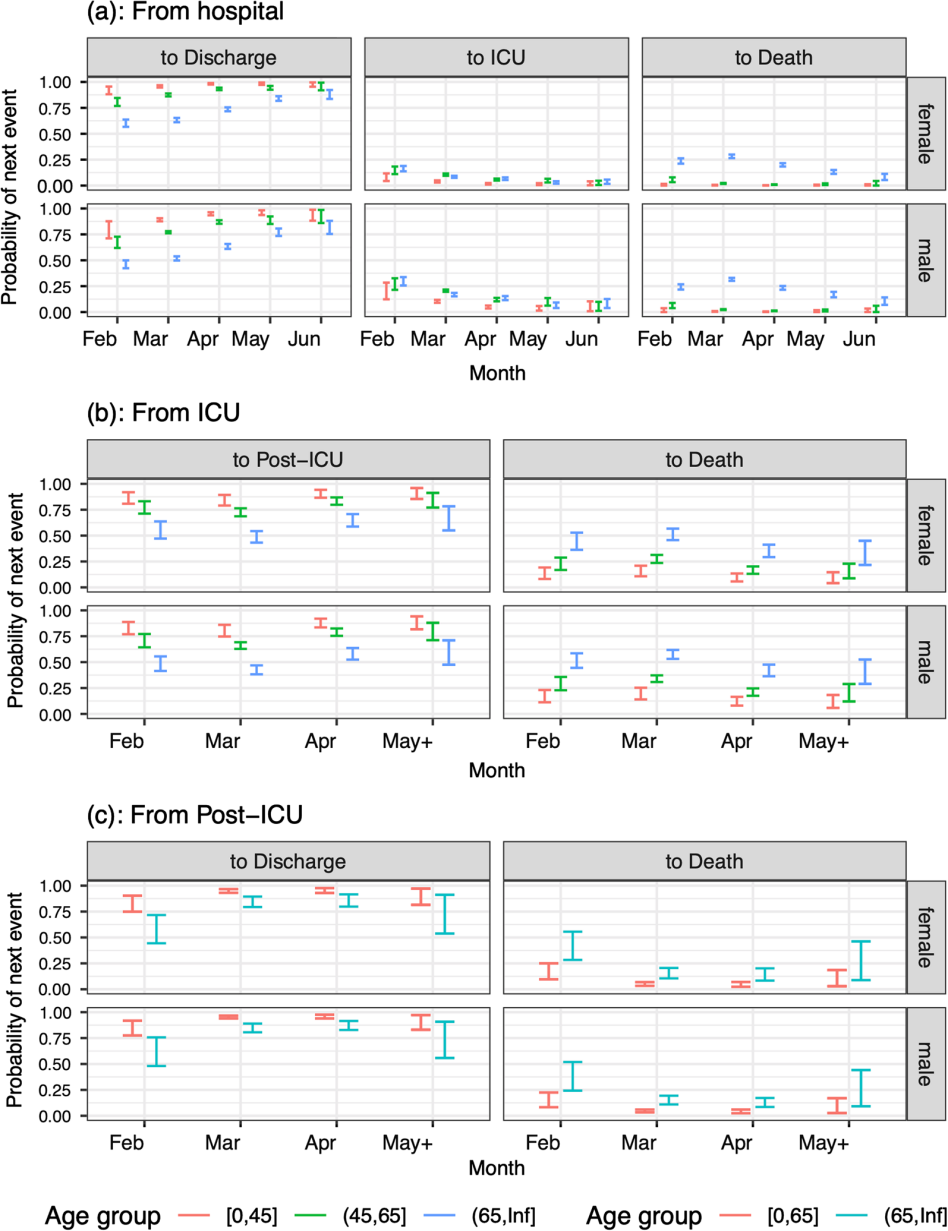
Predicted probabilities (95% confidence intervals) of moving from **a** hospital to next events; **b** ICU to next events; **c** a post-ICU stay to next events; by age, sex and month of admission. All other covariates are at their baseline if they are included in the selected model, i.e. patients in the largest capacity hospitals, without co-morbidities, who are neither care home residents nor healthcare workers

For most of the selected models for either the probabilities of or times to next events, age was an important covariate, whereas sex, if significant, had only small effect sizes. All results in this section concern patients with no co-morbidities in the largest capacity hospitals, who are neither care home residents nor healthcare workers. The probability of ICU admission was larger in men than women and in the two older age groups than the youngest; and reduced with month of admission (ORs April 0.644 95% confidence interval [0.553–0.750]; May 0.230 [0.143–0.372]; and June 0.247 [0.120–0.5080] relative to March), see Fig. 3(a)). The probability of death without ICU admission was much larger in those aged 65 + than those younger, falling from a peak of around 30% in March to 10% or less in June (Fig. 3a). The probability of death in ICU increased with age and was slightly larger for men than for women (Fig. 3b), falling from a high of 57.4% in men aged 65 + (49.8% in women aged 65 +) in March to 38.3% (31.4%) in May & June. The probability of death post-ICU is larger in the 65 + age group than those younger; and is similar in men and women (Fig. 3c).

**Fig. 4:**
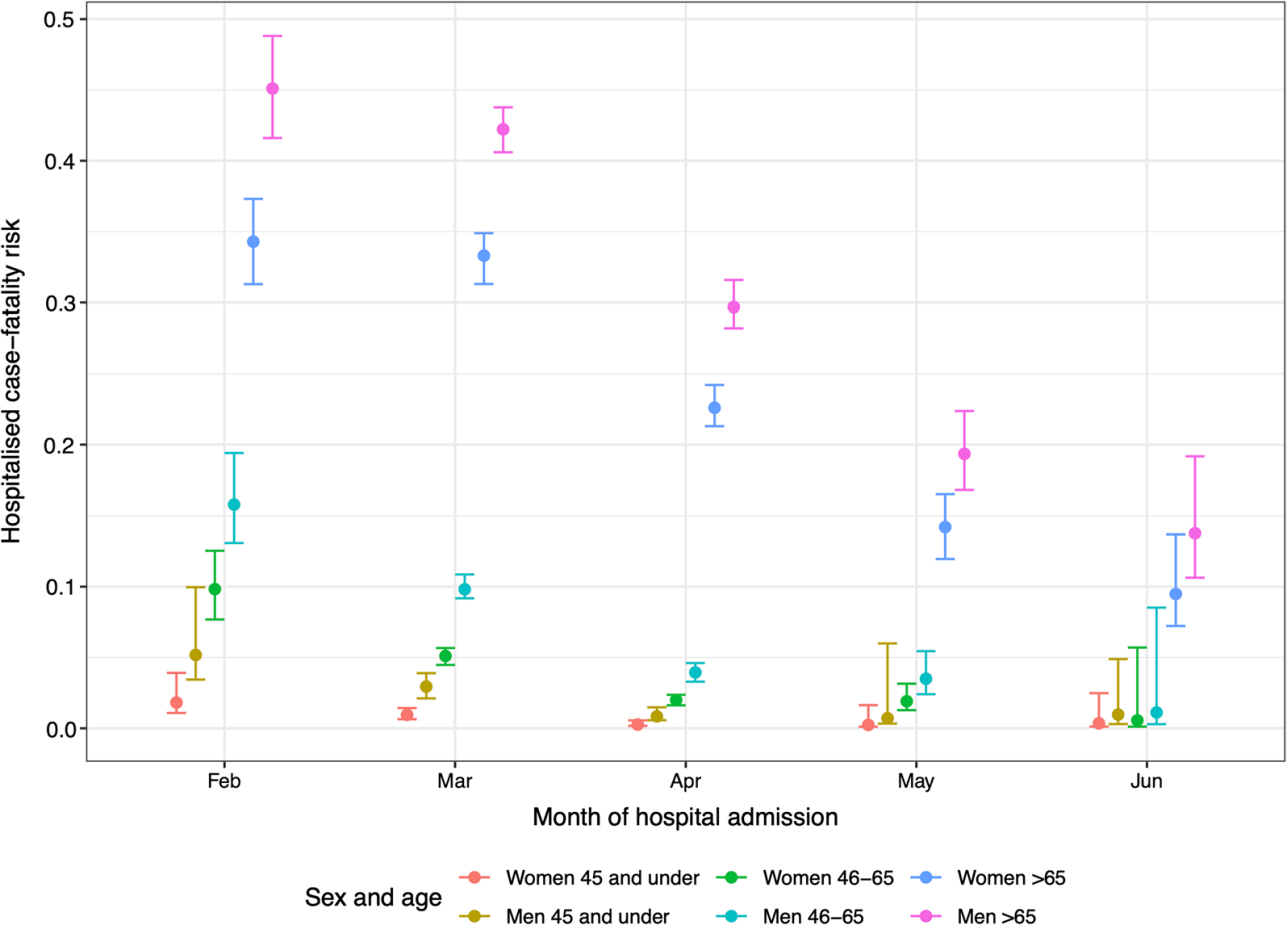
Estimated hospital fatality-risk by age, sex and month of admission, for patients in the largest capacity hospitals, without co-morbidities, who are neither care home residents nor healthcare workers

The probabilities of death from each stage of hospital care combine to obtain the hospital fatality-risk, averaged over pathways through hospital, as shown in Fig. 4. Further summaries of the probabilities of each pathway through hospital and lengths of stay by pathway through hospital are shown in Additional file 1: Appendix S4.

**Fig. 5:**
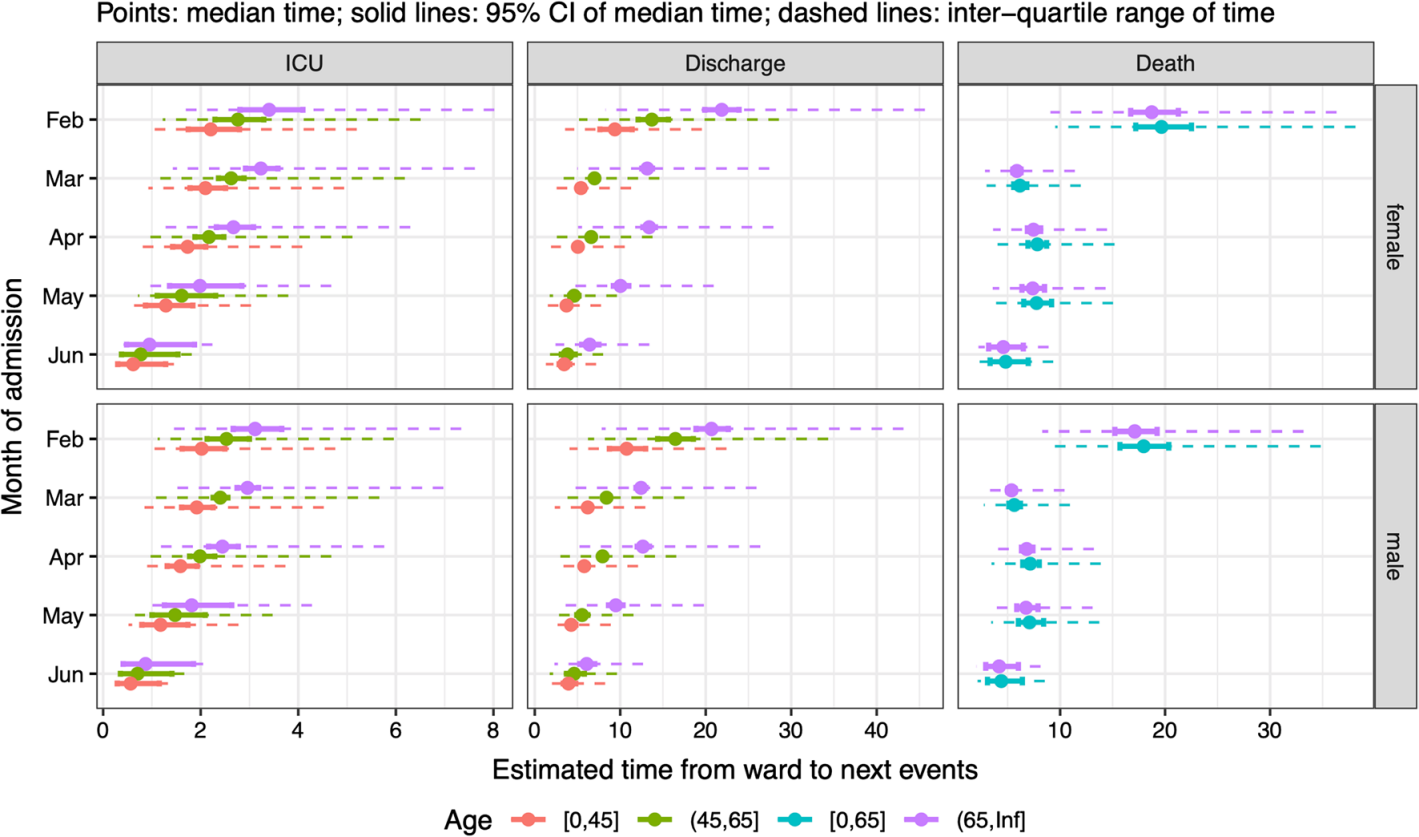
Summaries of distributions of time from admission to a hospital ward to next events

The median time from hospital admission to either an ICU admission or a discharge increased with age, did not substantially differ by sex, and decreased with month of admission (Fig. 5, first two columns). The median time to ICU admission reduced from around 3 days in February to less than a day in June; the median LoS for a patient discharged without ICU admission decreased from more than 20 days in 65 + year-olds in February to 6 days in the same group in June. LoS among patients not admitted to ICU who died did not vary much by age or sex, and was only substantially larger, but also more uncertain, for admissions in February (around 15–25 days) than for later admissions (less than 10 days, Fig. 5, 3rd column).

**Fig. 6:**
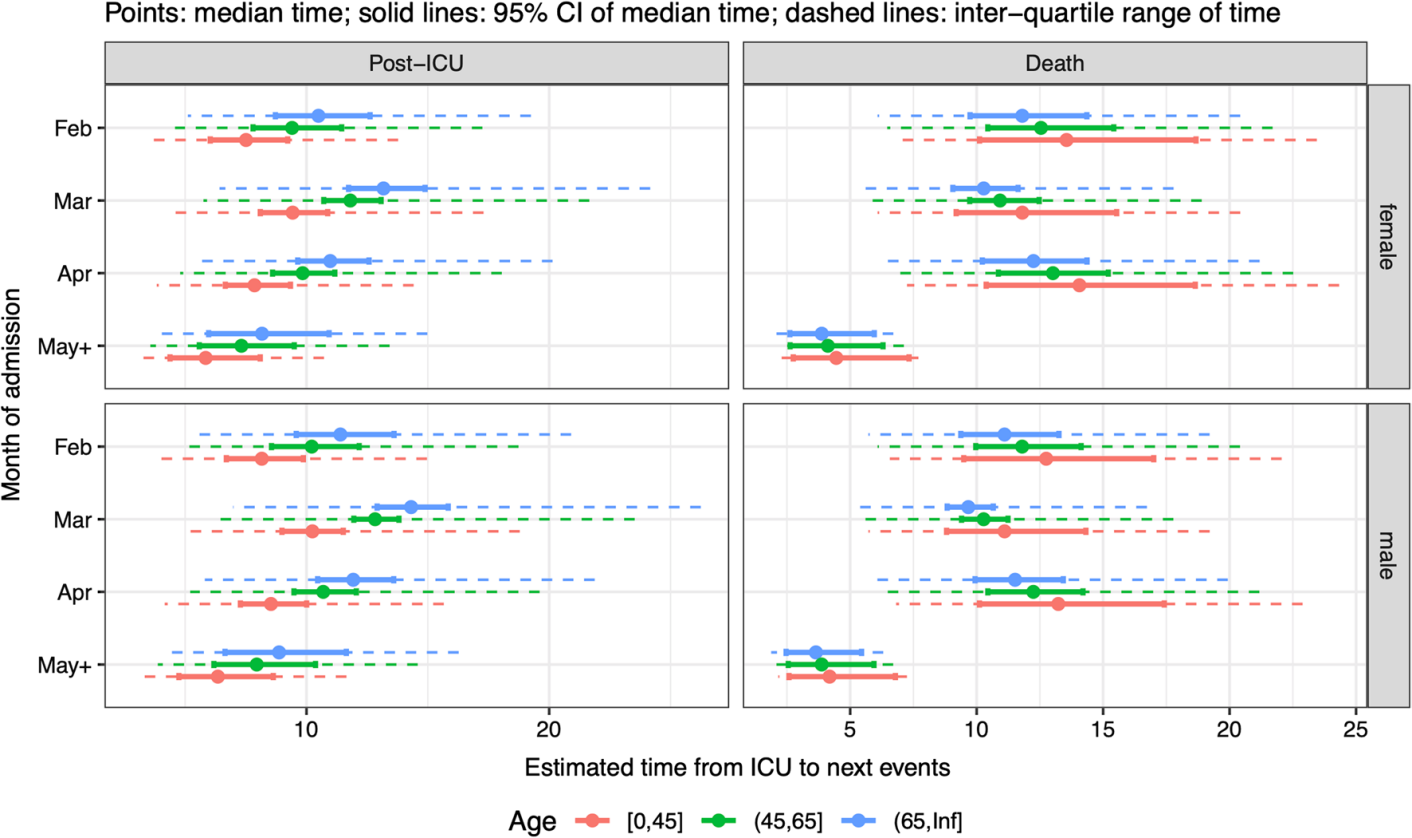
Summaries of distributions of time from ICU admission to next events

Median LoS in ICU did not differ substantially by sex; increased with age among survivors; decreased with month from a peak in March among survivors (Fig. 6 left column); decreased with age; and did not vary substantially with month for non-survivors, other than a decrease in May–June compared to earlier months (Fig. 6 right column).

**Fig. 7:**
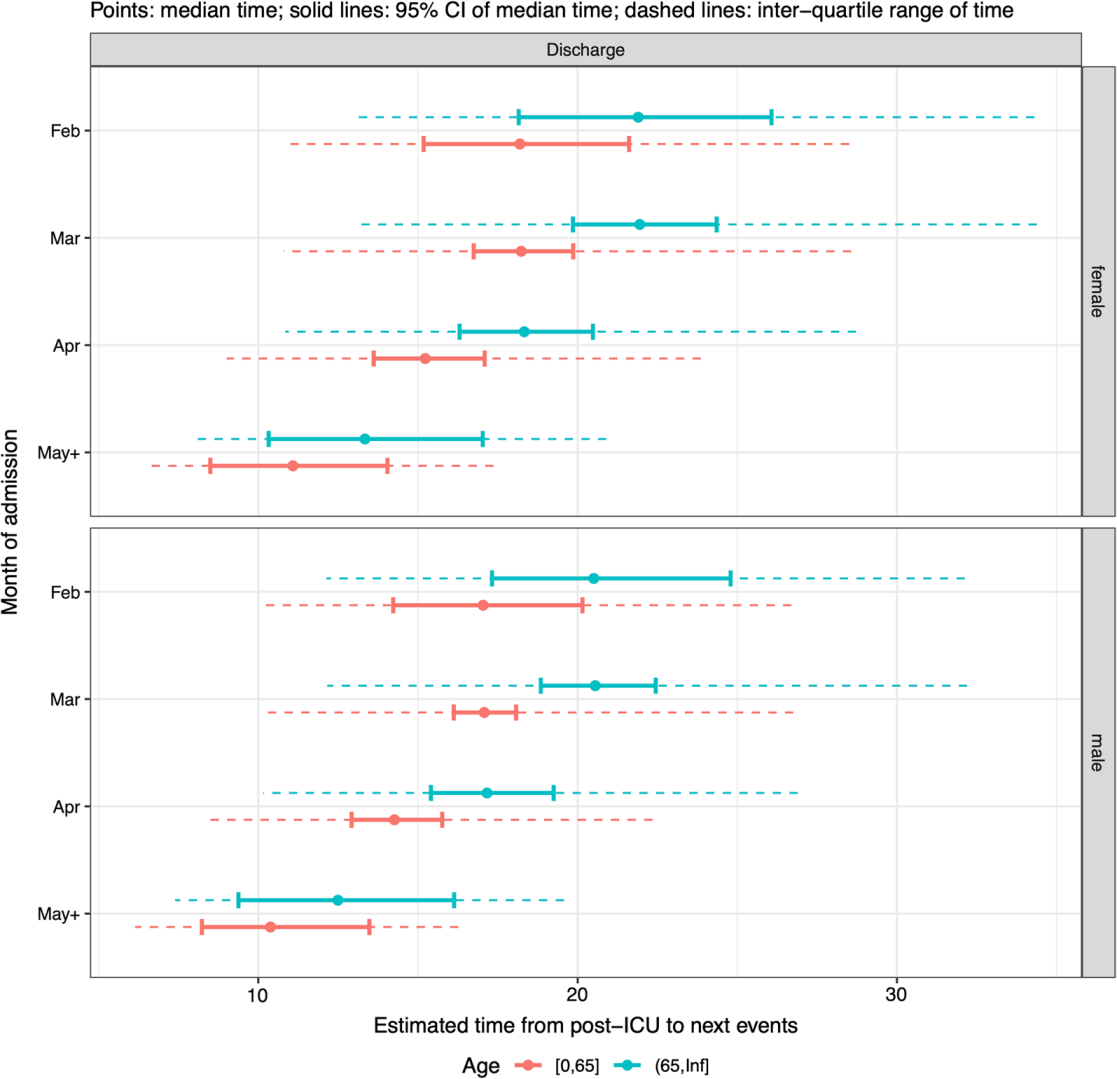
Summaries of distributions of length of stay in a post-ICU ward, among survivors

Median LoS post-ICU increased with age, was slightly longer in women than men, and decreased with month of admission, among patients who survived to discharge (Fig. 7). The number of deaths observed post-ICU was small, so month of admission, age and sex were not selected as significant covariates for post-ICU LoS among non-survivors.

### Care home residence

**Fig. 8:**
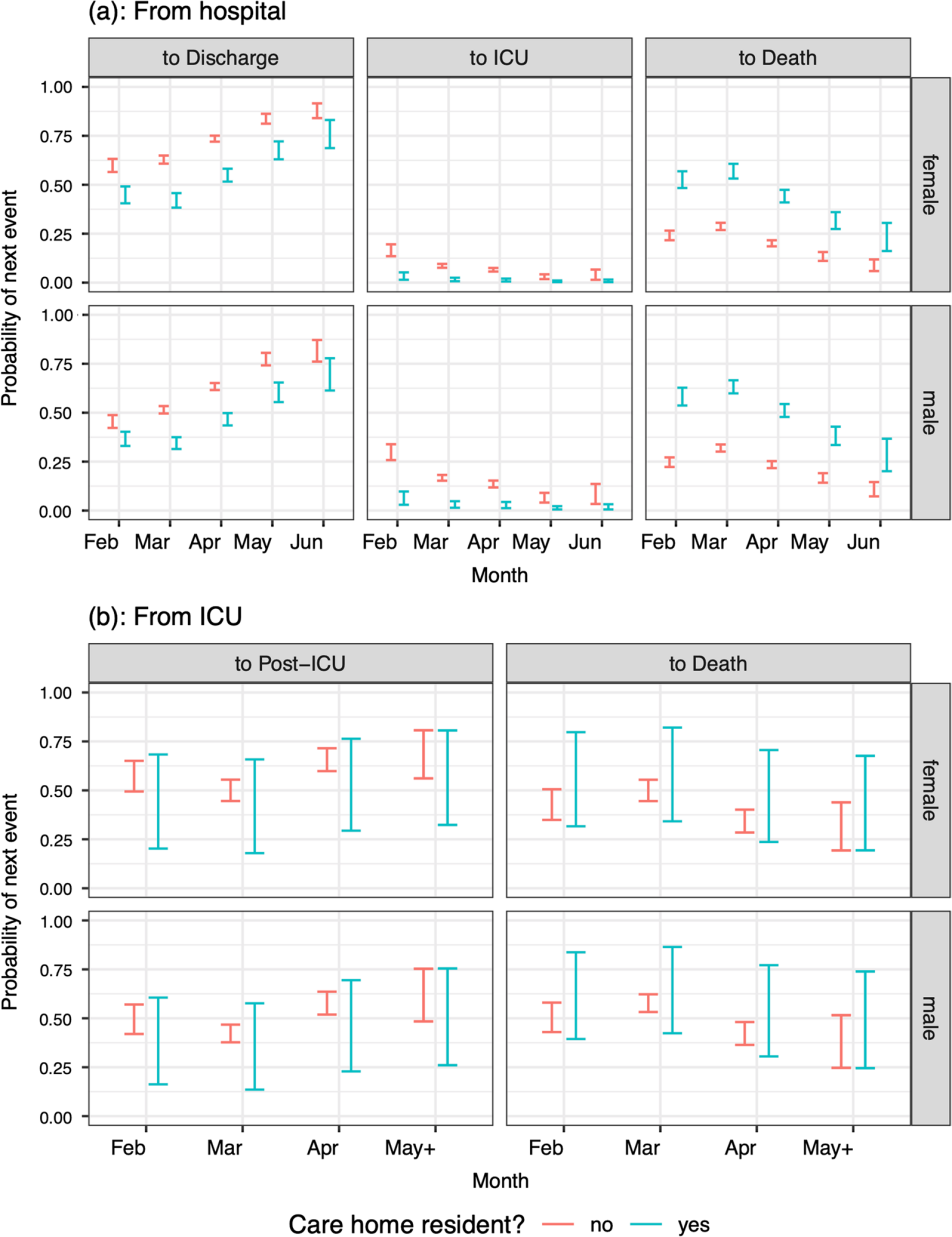
Predicted probabilities (95% confidence intervals) of moving from **a** hospital or **b** ICU to next events (columns), by sex (rows), month of admission (x-axis) and care home residency (colours), among patients aged 65 + in large hospitals, with no co-morbidities, and who are not healthcare workers

Being a care home resident aged 65 + was substantially associated with the probabilities of being admitted to ICU (odds ratio 0.251 [0.143–0.439] relative to non-care-home residents) and of dying without ICU admission (odds ratio 3.037 [2.698–3.419] relative to non-care-home residents), resulting in the predicted probabilities shown in Fig. 8a, right-hand two columns. Care home residency was less associated with the probability of survival from an ICU admission (Fig. 8b).

**Fig. 9:**
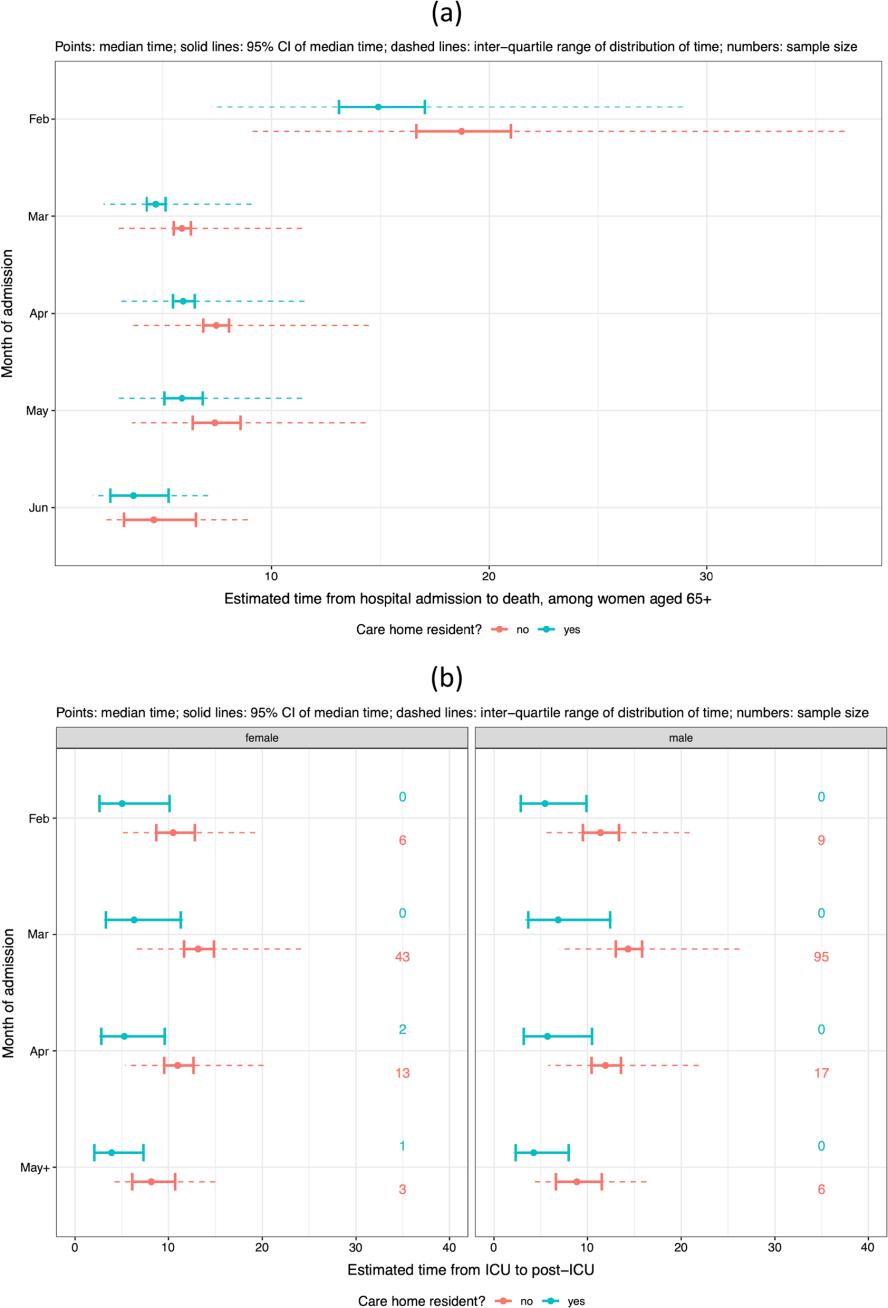
Estimated length of stay, by care home residence, sex and period of admission, in: **a** hospital for 65 + year-old women in large hospitals with no co-morbidities, who are not healthcare workers, and who die without ICU admission; **b** ICU for 65 + year-old patients in large hospitals with no co-morbidities, who are not healthcare workers, and who survive till a post-ICU stay. Note the small sample sizes and hence uncertain estimate of the median times for care home residents: although these are the sample sizes only for the baseline strata of other covariates, the estimates are also influenced by the sample sizes in non-baseline strata

Care home residence among 65 + year-olds was significantly associated with LoS (Fig. 9), substantially reducing both the hospital LoS for non-survivors not admitted to ICU (ETR 0.796, [0.736–0.860] relative to non-care-home residents) and the ICU LoS for survivors (ETR 0.480 [0.262–0.878] relative to non-care-home residents).

### Healthcare workers

**Fig. 10:**
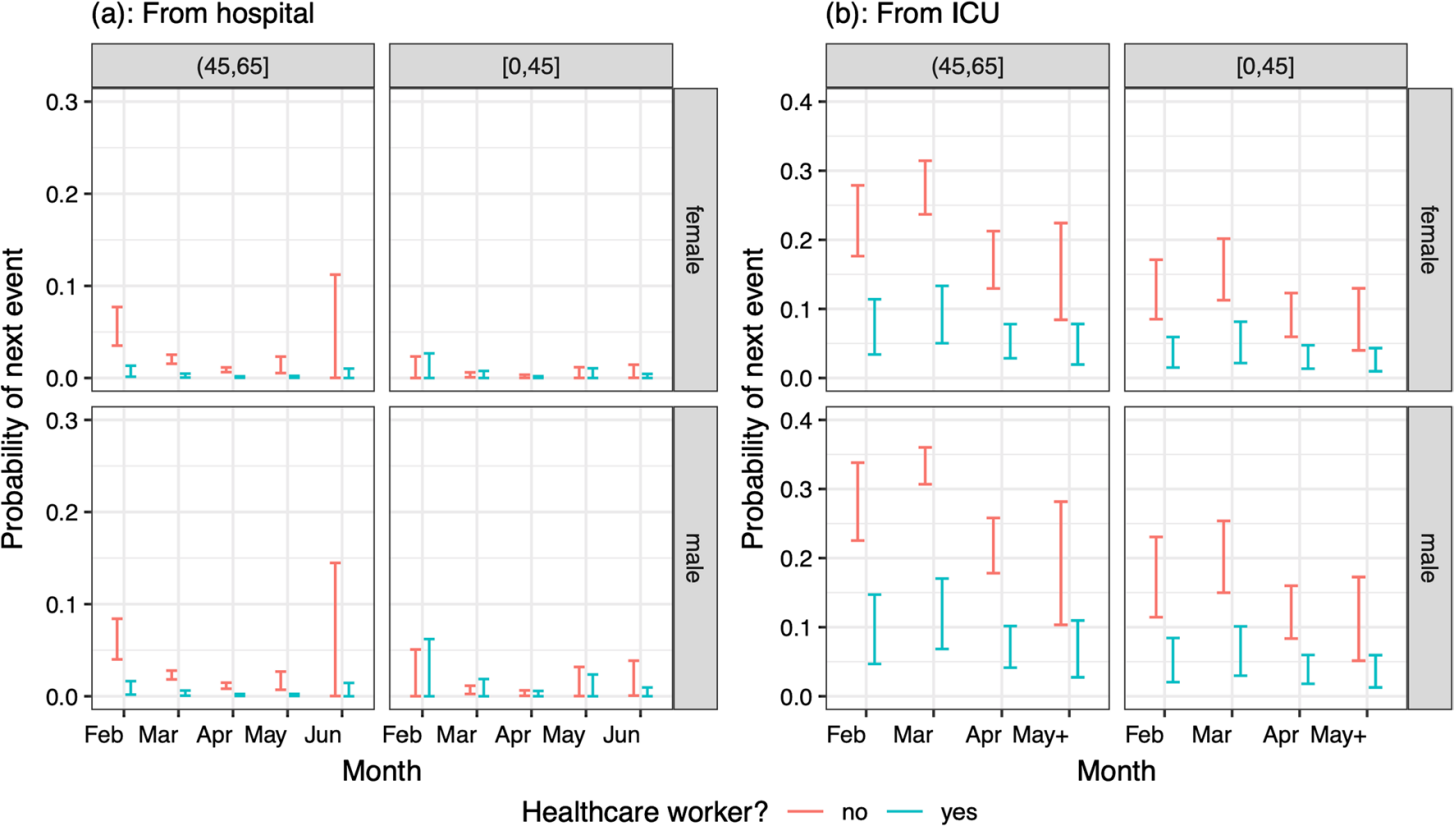
Predicted probabilities (95% confidence intervals) of death from either **a** hospital or **b** ICU, by age group (columns for ages less than 65), sex (rows), month of admission (x-axis) and healthcare worker status (colours), for patients with no co-morbidities in the largest hospitals, who are not care home residents

Being a healthcare worker aged 45–65 was slightly associated with a lower risk of death from hospital without ICU, particularly in the earlier months of admission (Fig. 10a). It is notable that being a healthcare worker in the youngest age group showed little difference in the risk of death from hospital without ICU compared to other patients. Healthcare workers had a significantly lower risk of death from an ICU admission (odds ratio 0.254 [0.143–0.453] relative to non-healthcare workers), resulting in predicted probabilities less than 10% in healthcare workers, compared to greater than 10% in other ICU patients (Fig. 10b).

**Fig. 11:**
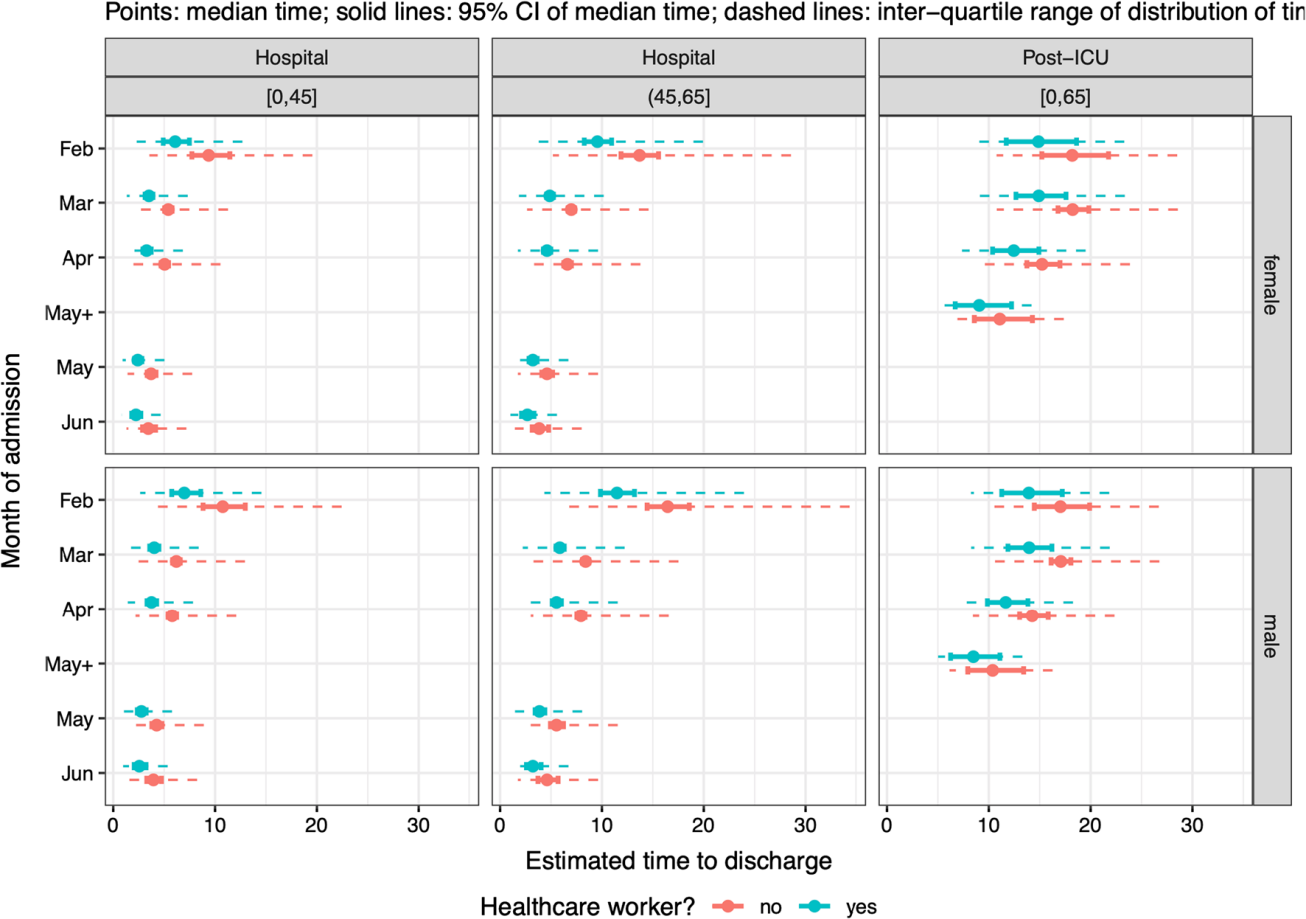
Summaries of distribution of time from either a hospital ward (columns 1 and 2) or a post-ICU stay (column 3) to discharge, by age group, sex, month of admission and healthcare worker status, among patients with no co-morbidities in the largest hospitals, who are not care home residents

Being a healthcare worker shortened the median LoS in hospital amongst survivors aged under 65 and not admitted to ICU (Fig. 11, first two columns). Healthcare workers were also significantly associated with a shorter LoS post-ICU for survivors aged under 65 (Fig. 11, third column) and for those aged 65 + (ETR 0.818 [0.702–0.953] relative to non-healthcare workers).

### Co-morbidities

**Fig. 12:**
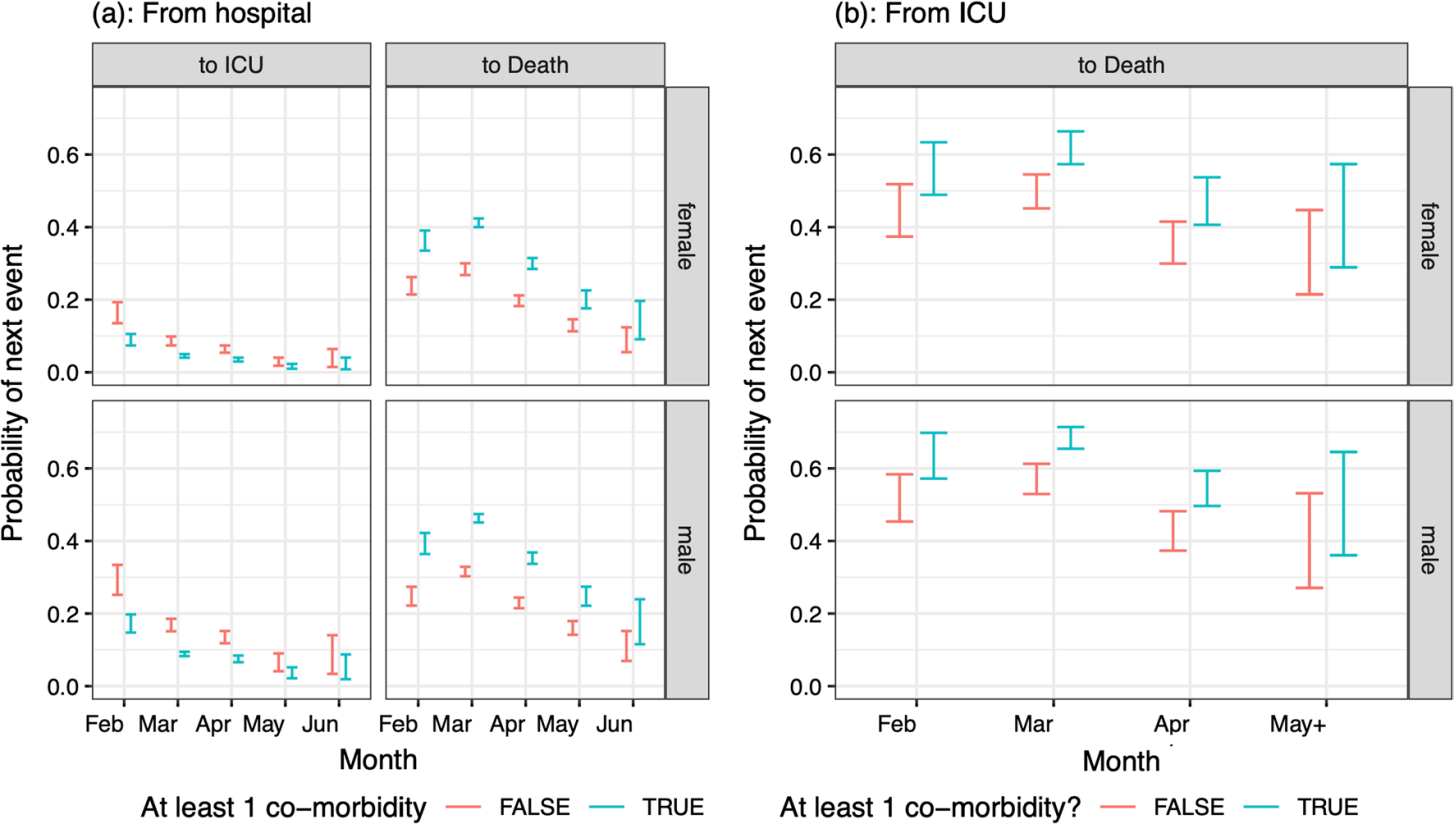
Predicted probabilities (95% confidence intervals) of moving from either **a** hospital admission or **b** ICU admission to next events, among patients aged 65 + in the largest hospitals, who are neither care home residents nor healthcare workers, by sex (rows), month of admission (x-axis) and co-morbidities (colours)

Having at least one co-morbidity was significantly associated with probabilities of next events among 65 + year-olds, although with small effect sizes: reducing the risk of ICU admission, but increasing the risk of death, from either a hospital ward or ICU (Fig. 12). There was a small but significant association of co-morbidity with LoS among survivors not admitted to ICU, with 65 + year-old patients with co-morbidities having a longer LoS than those with none (ETR 1.120 [1.071–1.172]). Co-morbidity was also slightly associated with ICU LoS among non-survivors, with a small but just significant effect size (65 + year-old patients have a shorter time to death, ETR 0.915 [0.837–1.000] relative to patients with no co-morbidities).

### Hospital bed capacity

**Fig. 13:**
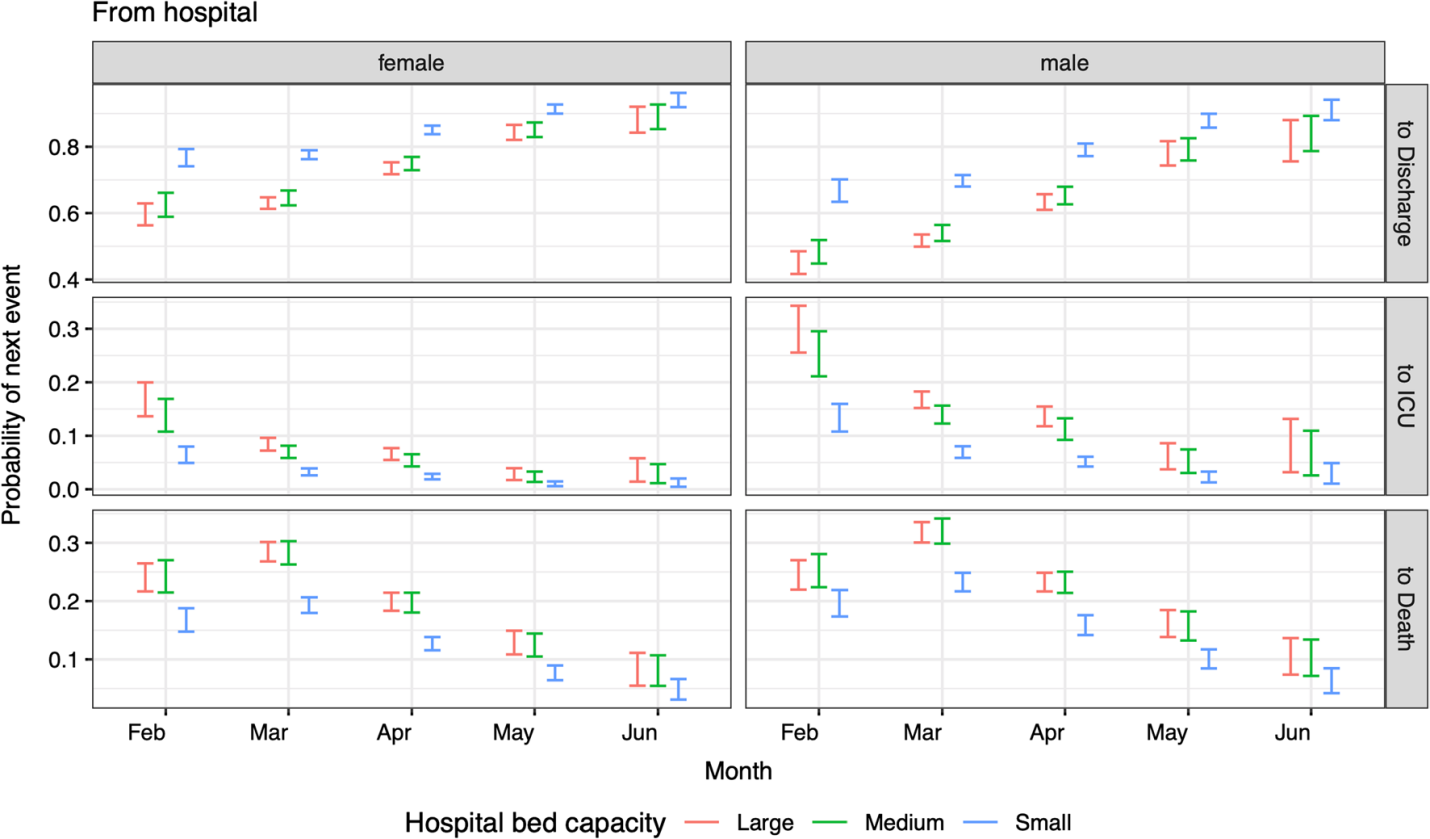
Predicted probabilities (95% confidence intervals) of moving from hospital admission to next events (rows) for patients aged 65 + with no co-morbidities and who are neither care home residents nor healthcare workers, by sex (columns), month of admission (x-axis) and hospital bed capacity (colours)

Grosso, Presanis et al. [8] found hospital bed capacity was significantly associated with both probabilities and times in univariable models. Here the association remained when adjusting also for patient characteristics. In fact, the effect size on probabilities of next events became larger, for those aged 65 + in the multivariably-adjusted model: the smallest hospitals have both the lowest risk of ICU admission (odds ratio 0.305 [0.254–0.367] relative to the largest hospitals) and the lowest risk of death without ICU admission (odds ratio 0.547 [0.507–0.590] relative to the largest hospitals) for this age group (Fig. 13). The risk of death in ICU, for all age groups, was also smaller in the smallest hospitals compared to the largest (odds ratio 0.270 [0.202–0.363] for 65 +). As previously noted [8], the lower risks of ICU-related events in smaller hospitals may, however, be an artefact of the smaller hospitals having less ICU capacity.

## Discussion and conclusions

This analysis is one of the first to demonstrate the association of key risk factors with hospital burden of COVID-19 in Regione Lombardia during the first wave, while correctly accounting for confounders, censoring and the competing risks of different pathways through hospital. To our knowledge, similar studies have been conducted in England, investigating trends in severe outcomes for hospitalised COVID-19 cases [6, 7]. Their findings are comparable to ours in terms of direction and magnitude of the investigated associations; nevertheless, our findings provide important confirmation of these associations, particularly given the differences between the Italian and the English health systems. We estimated a steady decrease in the risks of severe events such as ICU admission and mortality. Lengths of stay in hospital among survivors who are eventually discharged have reduced over time. In contrast, there is less evidence of any change in the lengths of stay for non-survivors. Our hypothesis is that the decreasing risks of severe events and lengths of stay by month of admission reflect a combination of factors: (1) improvement in clinical knowledge, patient management and treatment [17]; (2) improvements in the surveillance system, which allowed identification of infected individuals earlier in their infection, resulting in better management of their disease [18]; (3) the decrease in infection rates over the months following the introduction of the strict lockdown [19]. The implication of these results in terms of specific risk factors are discussed below.

Consistently with other studies [20], risks of ICU admission and fatality increase with age group, presence of comorbidities, and are generally higher for men than for women. Similarly, lengths of stay in hospitals increase with age for survivors, but slightly decrease with age for non-survivors, although the differences are small for non-survivors. This latter finding may be because patients who die may be fragile in terms of unobserved confounders not already captured by the co-morbidities and other covariates, so that younger age does not confer any substantial advantage. Sex appears to be only marginally associated with length of stay.

Although risk of being infected with SARS-CoV-2 is three-fold higher for healthcare workers than in the general population [21], and this group has an elevated prevalence of prior infection [22, 23], they appear more protected in terms of shorter lengths of stay in ICU and hospital, among individuals under 65 years of age; and to have overall significantly lower risk of death in ICU. Working-age healthcare workers have similar hospital-fatality risks to other patients, particularly in the 0–45 year-old group, but have lower ICU-fatality risks. These findings are consistent with other studies in healthcare workers during the MERS epidemic [24, 25]. Having easy access to the hospital and clinicians and being physically present for long shifts may have been an advantage for those that fell ill, allowing early diagnosis, close monitoring and timely treatment. Also, we cannot rule out that part of the protection may be attributed to some sort of empathetic “comrade effect”. Further studies are needed to ascertain this protective association.

COVID-19 outbreaks in care homes have been hotspots of the pandemic in Lombardia. In April, hospitals overwhelmed with in-patients, and required to provide more and more beds, were allowed to discharge patients still in need of low-intensity care to suitable care homes [26]. Debate is ongoing about whether these discharges might have increased transmission in this setting, although a survey from the Italian National Institute of Health on care homes in Italy highlighted that deaths in Lombardia care homes occurred mostly during 16^th^-31^st^ March 2020 [27]. Burton et al. [28] reported that 4% of deaths among care home residents in Scotland occurred in hospitals, whereas the European Center for Disease Control [29] reported that in Belgium, the corresponding proportion was 28%. We have shown that being a care home resident was associated with a significant risk of not being admitted to ICU and dying in hospital without going through ICU. The largest effect sizes were seen for care home residents in the 65 + age group, who are at greater risk of death, and for whom lengths of stay are shorter, whether they survive or not. Note that the numbers of care home residents observed to be admitted to hospital as a confirmed COVID-19 case in the first few weeks of the epidemic were small (24 in February, 344 in March), and of these, none were admitted to ICU. These small sample sizes result in uncertain estimates of the probability of ICU admission among care home residents in these months (around 3–5% in 65 + year olds, compared to 10–30% in 65 + year old non-care-home residents), that were therefore influenced by the downward trend observed in non-care-home residents (Fig. 8a). More care home residents were admitted in April, two months after the start of the pandemic, when hospitals were already overwhelmed. Our hypothesis is that these extremely fragile patients reached the hospital in too severe condition to be considered eligible for ICU and many also died before being admitted to ICU. A potential contribution to the elevated risk for care home residents may be that in the relatively wide 65 + age group, care home residents have an older age distribution than non-residents. Further studies are needed to investigate this sensitive issue.

The multi-state modelling approach employed allows comprehensive and simultaneous estimation of all hospital case-severity risks and lengths of stay of interest, under some assumptions. The dataset used is relatively complete and is a comprehensive census of confirmed cases hospitalised during the first wave, so is thought to be representative of hospitalised cases in the region. We have, however, assumed missingness is ignorable for some key covariates and for final outcomes [10] and that exclusions (Additional file 1: Appendix S1, Figure S1) have not biased results in any way. However, although uni- and bi-variable models were not sensitive to the ignorable missing outcomes assumption [10], sensitivity for the multivariable analyses has not been assessed, due to computational complexity. A selection bias due to only tested and confirmed cases being included in the register could in theory be present, since untested and undiagnosed cases would more likely have been infected in the early part of the first wave when testing was not yet widely available. However, such cases were also more likely to have been only mildly symptomatic or asymptomatic, and therefore unlikely to have been hospitalised. We thus believe such selection is unlikely to have biased our analyses based on the hospitalised subset of cases, since the vast majority of hospitalised cases will have been tested and confirmed as a case. Risk factors potentially associated with hospital burden and severity that were not available in the register of confirmed cases were ethnicity, socio-economic deprivation and severity of illness at hospital admission, so we were not able to assess any such association.

Further work is underway to apply a similar multi-state model to the subset from the Milano Local Healthcare Agency district, where good information is available on symptom onset dates, to understand the burden in the community, such as the proportion of symptomatic cases that are hospitalised or who die in the community, as well as the corresponding distributions of times to these events. Such an analysis, combined with the within-hospital analysis here, will provide further crucial evidence on the burden of COVID-19 in Lombardia, in particular the overall symptomatic case-fatality risk.

## Supplementary Information

**Additional file 1. **Additional figures and tables.

## Data Availability

The pseudo-anonymised data that support the findings of this study are available from the General Directorate of Welfare of Regione Lombardia but restrictions apply to the availability of these data, which were used under license for the current study, and so are not publicly available. Data are however available from the authors upon reasonable request and with permission of the COVID-19 Research Committee of the General Directorate of Welfare of Regione Lombardia.

## Abbreviations

AIC: Akaike’s Information Criterion
CI: Confidence Interval
COVID-19: Coronavirus Disease 2019
ETR: Expected Time Ratio
ICU: Intensive Care Unit
IQR: Interquartile Range
LoS: Length of Stay
PCR: Polymerase chain reaction
SARS-CoV-2: Severe Acute Respiratory Syndrome-Coronavirus-2
