# Supplementary material for: Risk factors associated with severe hospital burden of COVID-19 disease in Regione Lombardia: a cohort study
Source: BMC Infect Dis. 2021 Oct 7;21:1041. doi: 10.1186/s12879-021-06750-z (PMC8496148; doi:10.1186/s12879-021-06750-z)
Supplement: Supplementary file 1 — Additional file 1. Additional figures and tables. [file 12879_2021_6750_MOESM1_ESM.pdf]

Supplementary Material
Risk factors associated with severe hospital burden of COVID-19 disease in Regione Lombardia: a cohort study
Presanis, Kunzmann et al

A.1 Lombardia Covid-19 Regional Database

The Lombardia Covid-19 Regional Database is an id-linked pseudo-anonymised database of all individuals who became cases confirmed by RT-PCR testing of their oropharyngeal swabs during the first wave of the epidemic up till 17th July 2020. Eventual hospital admission was recorded and matched to an individual's ID, as well as hospital discharge or death. The hospitalised subset analysed here was selected from the full database based on the exclusions shown in Figure A.1.

The dataset is relatively complete for most covariates of interest. Non-missing data on co-morbidities (pre-existing medical conditions), among respiratory, cardio-vascular, metabolic or oncological categories, are used to define a co-morbidity flag: existence of at least one co-morbidity corresponds to a flag equal to 1; whereas missing information on co-morbidity corresponds to a flag equal to 0. The symptoms covariate is missing for the majority (64%) of patients, so is not considered further. District is missing for 5.7% of patients, but is not of primary interest in this analysis, so is also not considered further. Hospital size is defined in terms of hospital bed capacity as small, medium or large, depending on numbers of both hospital and ICU beds (Table A.1).

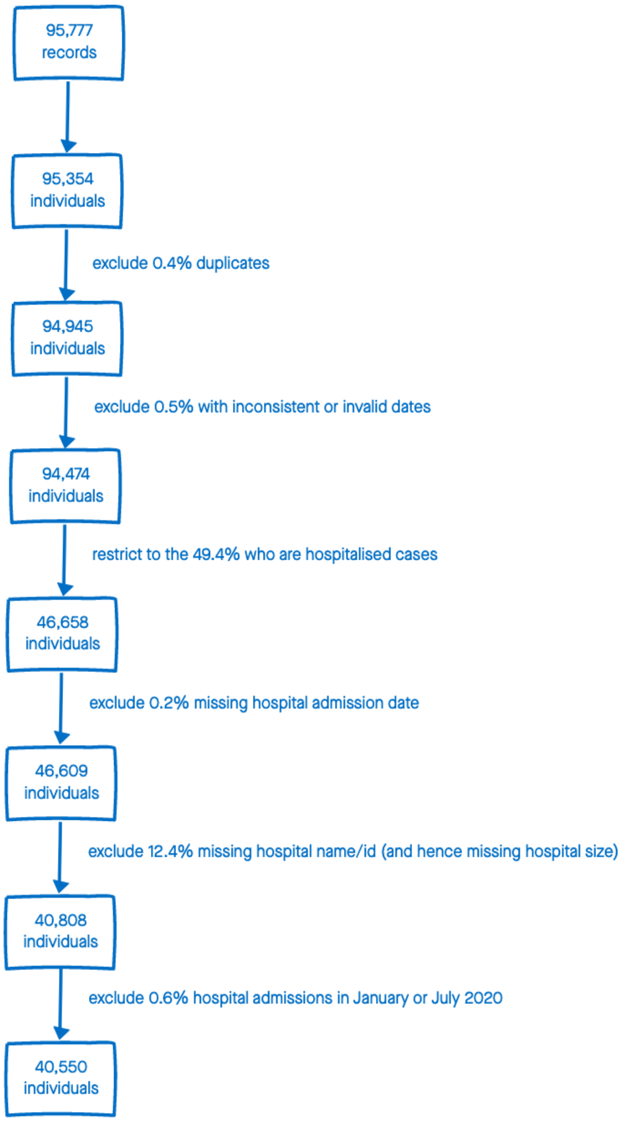

Figure A.1: Flowchart of data exclusions in the Covid-19 Regional Database to obtain the hospitalised cohort.

			Number of ICU beds		
			**Small**	**Medium**	**Large**
			**[0,1]**	**(1,6]**	**(6,71]**
**Number of hospital beds**	**Small**	**[0,88]**	Small	Small	Medium
			52	1	0
	**Medium**	**(88,191]**	Small	Medium	Large
			27	18	5
	**Large**	**(191,1063]**	Medium	Large	Large
			5	18	27

Table A.1: Definition of hospital bed capacity. Both the total number of beds (rows) and the number of ICU beds (columns) are categorised into small, medium and large by quantiles over the number of hospitals of the respective numbers of beds (33% in each category for total hospital beds, 50% in the small category and 25% in the medium and large categories for ICU beds). These definitions for total and ICU beds are then combined into a single "hospital bed capacity" variable as shown in the table cells, resulting in 80 small hospitals, 23 medium hospitals and 50 large hospitals.

A.2 Multivariable models - probabilities of next events

The selected covariates are summarised in Table A.2. Covariate effects on each of the transition probabilities are given in Tables A.3 to A.5 and the corresponding predicted probabilities for patients with particular covariate levels are shown in Figures A.2 to A.10. Note that the solid lines give predicted probabilities, but are not continuous through calendar time: the lines represent 'joining the dots' of the predictions for each month of admission, so as to differentiate between the predictions and the observations. The dots, on the other hand, represent the observed proportions.

Transition probabilities	Distribution	Selected covariates	Log-likelihood	AIC
Hospital to ICU or death	Multinomial	age group * (sex + month of admission + co-morbidity + care home resident + healthcare worker + hospital bed capacity)	-27,388	54,907
ICU to death	Binomial	age group + sex + month of admission + co-morbidity + care home resident + healthcare worker + hospital bed capacity	-2,264	4,551
Post-ICU to death	Binomial	age group + sex + month of admission + co-morbidity	-524	1,062

Table A.2: Summary of selected covariates for each regression, based on minimum AIC and likelihood ratio tests. The star (*) notation denotes both main effects and interactions.

A.2.1 Hospital admission to discharge without ICU admission, ICU admission, or death without ICU admission

The selected multinomial logistic regression model from hospital ward to the next event includes main effects from all seven covariates and interactions of each covariate with age. The odds ratios corresponding to the main effects, i.e. at the baseline level of each of the other 6 covariates, are given in Table A.3. Predicted vs observed probabilities of moving from hospital ward to either discharge, ICU or death are shown in Figures A.2 to A.5, for selected covariates. Most effects are significant, with age group, sex, care home residency and month of hospital admission having the largest effect sizes. The interactions of age with month of admission, care home residency and co-morbidity are all important, with co-morbidity only having a visible effect in the 65+ age group (Figure A.4). Compared to March, the proportion of individuals moving from hospital ward to either death or ICU is decreasing with calendar month.

Transition to	Covariate level	OR at baseline	95% CI		p
ICU	Age group [0,45]	0.886	0.728	1.078	0.227
	Age group (45,65]	0.286	0.201	0.406	0.000
	Sex male	2.425	2.140	2.748	0.000
	Month of admission Feb	1.975	1.617	2.413	0.000
	Month of admission Apr	0.644	0.553	0.750	0.000

	Month of admission May	0.230	0.143	0.372	0.000
	Month of admission Jun	0.247	0.120	0.508	0.000
	At least 1 co-morbidity	0.608	0.537	0.690	0.000
	Care home resident	0.251	0.143	0.439	0.000
	Healthcare worker	0.813	0.379	1.745	0.596
	Hospital capacity Medium	0.786	0.668	0.924	0.004
	Hospital capacity Small	0.305	0.254	0.367	0.000
Death	Age group [0,45]	0.049	0.038	0.064	0.000
	Age group (45,65]	0.003	0.001	0.010	0.000
	Sex male	1.357	1.281	1.437	0.000
	Month of admission Feb	0.875	0.764	1.002	0.054
	Month of admission Apr	0.595	0.552	0.641	0.000
	Month of admission May	0.336	0.281	0.402	0.000
	Month of admission Jun	0.189	0.125	0.285	0.000
	At least 1 co-morbidity	1.679	1.552	1.816	0.000
	Care home resident	3.037	2.698	3.419	0.000
	Healthcare worker	0.057	0.014	0.235	0.000
	Hospital capacity Medium	0.965	0.890	1.046	0.385
	Hospital capacity Small	0.547	0.507	0.590	0.000

Table A.3: Estimated odds ratios for covariate effects on the probability of moving from a hospital ward to either ICU or death. The probability of moving from a hospital ward to discharge is the complement, 1 minus the other two probabilities. Note only main effects are shown here, i.e. the odds ratios at the baseline value of each other covariate. Interactions were included in this model, but are not shown for brevity: interactions should be combined with main effects to obtain effects at non-baseline values of each covariate.

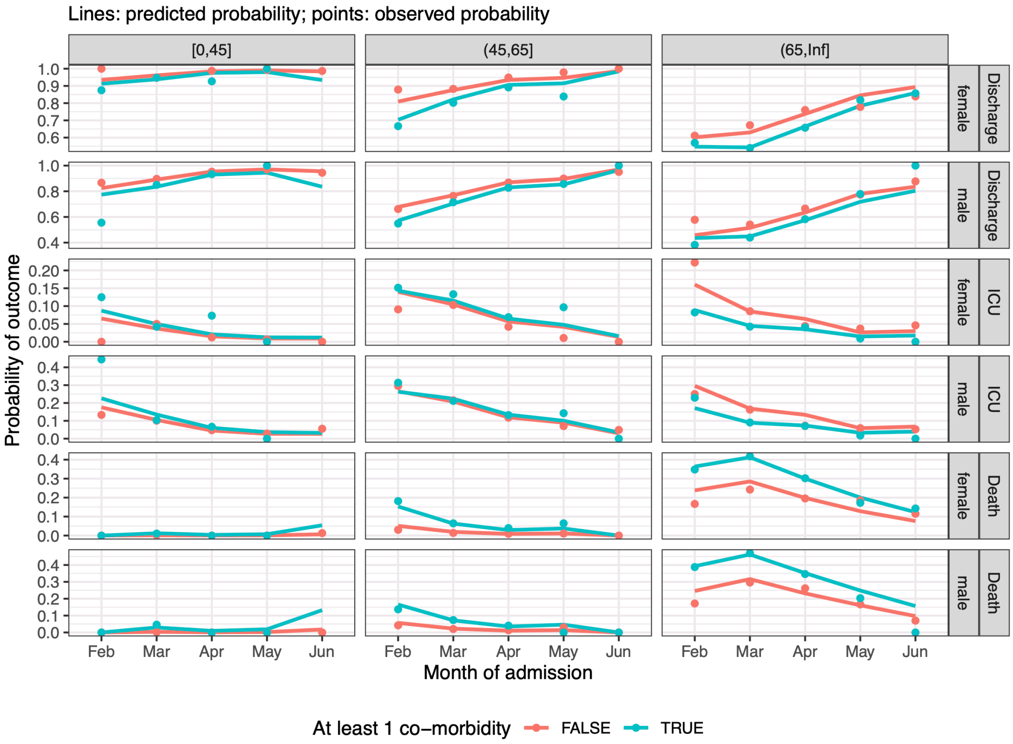

Figure A.2: Predicted vs observed probabilities of moving from a hospital ward to either discharge, ICU or death, by age, sex, month of admission and co-morbidity. All other covariates are set to their baseline values, i.e. these probabilities are for patients who are not care home residents, not healthcare workers, and are in a large hospital.

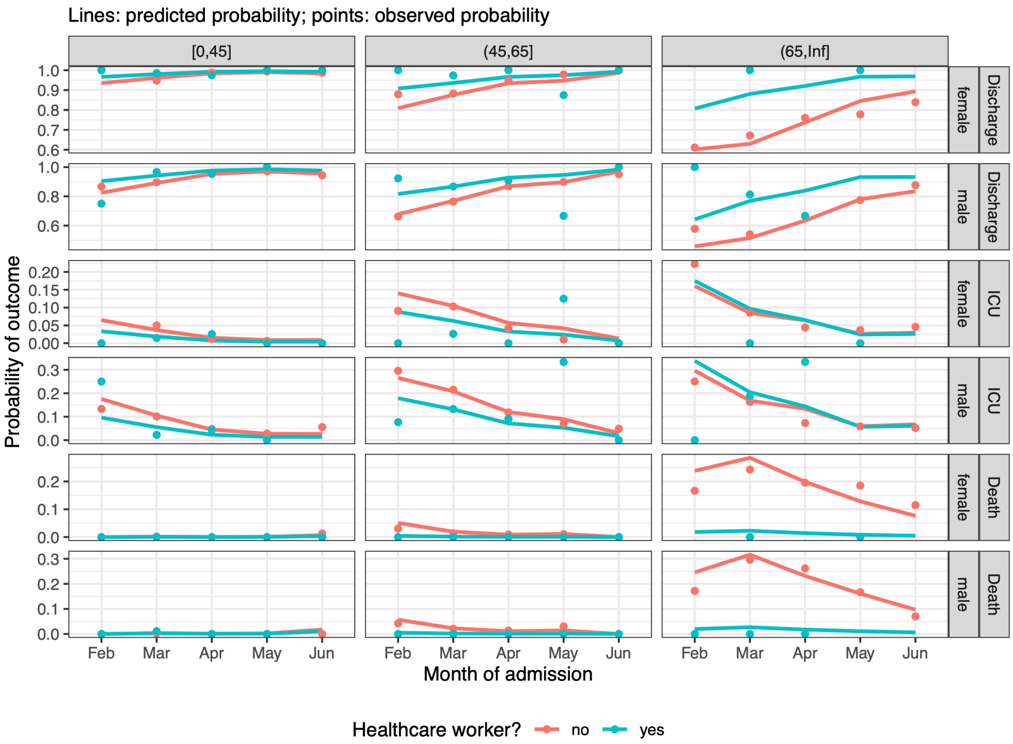

Figure A.3: Predicted vs observed probabilities of moving from a hospital ward to either discharge, ICU or death, by age, sex, month of admission and healthcare worker status. All other covariates are set to their baseline values, i.e. these probabilities are for patients who have no co-morbidity, are not care home residents, and are in a large hospital.

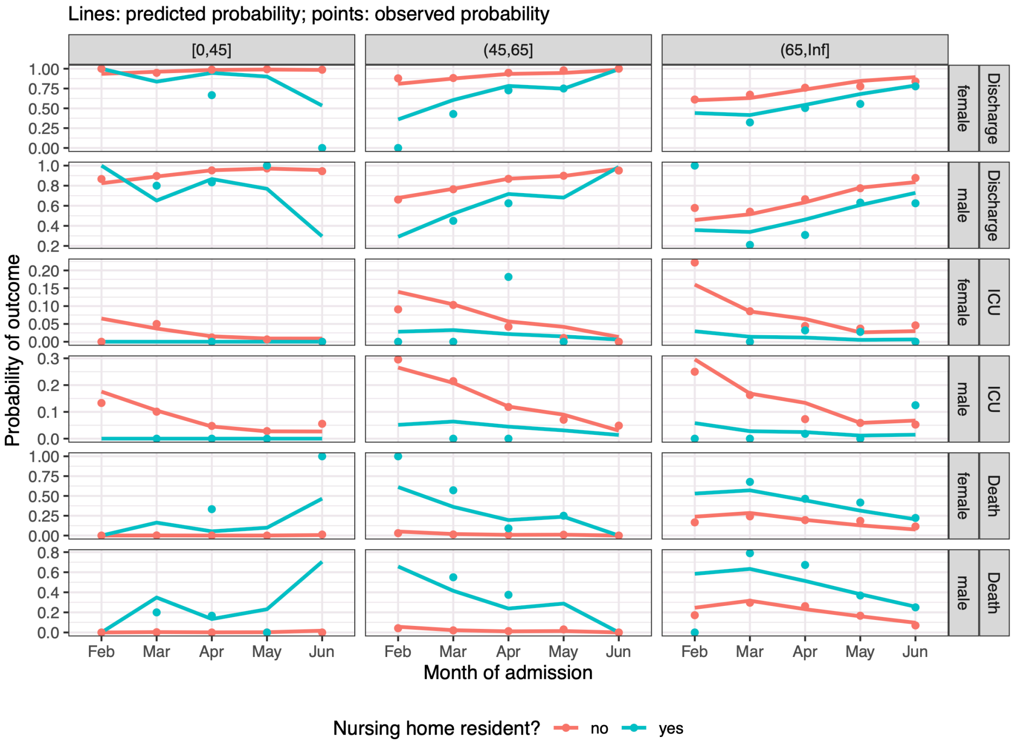

Figure A.4: Predicted vs observed probabilities of moving from a hospital ward to either discharge, ICU or death, by age, sex, month of admission and care home residency. All other covariates are set to their baseline values, i.e. these probabilities are for patients who have no co-morbidity, are not healthcare workers, and are in a large hospital.

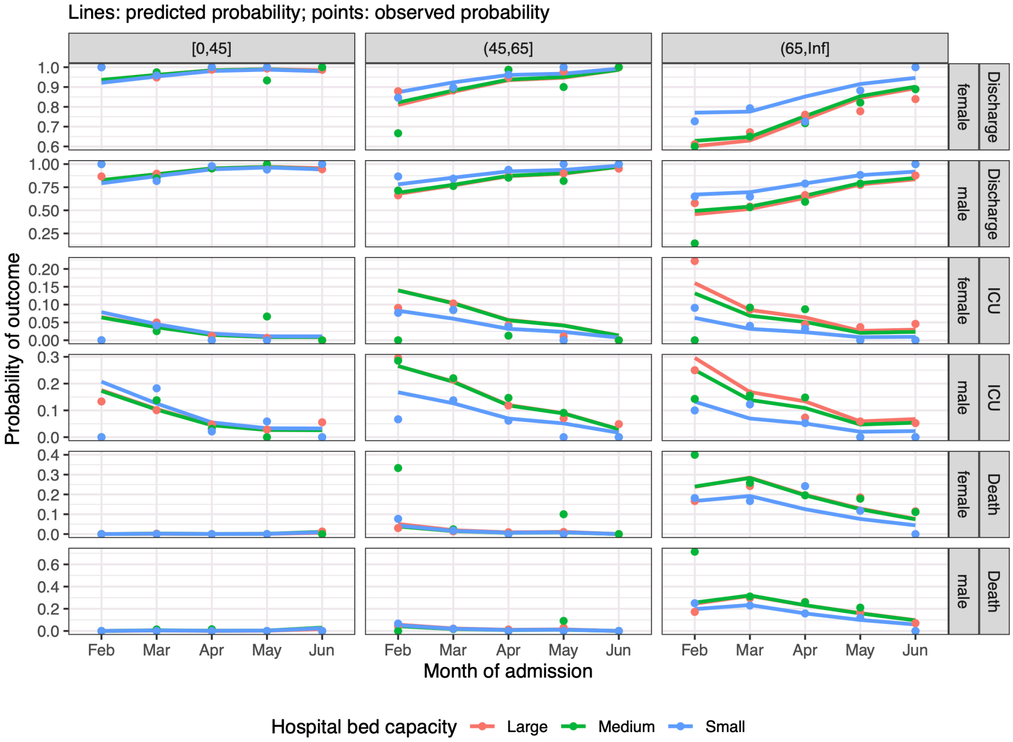

Figure A.5: Predicted vs observed probabilities of moving from a hospital ward to either discharge, ICU or death, by age, sex, month of admission and hospital bed capacity. All other covariates are set to their baseline values, i.e. these probabilities are for patients who have no co-morbidity and are not care home residents nor healthcare workers.

A.2.2 ICU admission to post-ICU stay or death

As there are so few transitions from ICU to next events where the month of admission is June, May and June are combined into a single covariate level for the purposes of this binomial logistic regression model. From ICU to death (Table A.4), the selected model includes all seven main effects, but no interactions. Age, healthcare worker status and hospital bed capacity have the largest

effects. The effect of calendar month relative to March on this transition has a less clear pattern over time than for the transitions from hospital ward.

Transition to	Covariate level	OR at baseline	95% CI		p
Death	Age group [0,45]	0.370	0.319	0.429	0.000
	Age group (45,65]	0.182	0.126	0.261	0.000
	Sex male	1.359	1.140	1.619	0.001
	Month of admission Feb	0.787	0.587	1.053	0.107
	Month of admission Apr	0.531	0.426	0.662	0.000
	Month of admission May-Jun	0.460	0.245	0.866	0.016
	At least 1 co-morbidity	1.617	1.394	1.877	0.000
	Care home resident	1.609	0.588	4.408	0.355
	Healthcare worker	0.254	0.143	0.453	0.000
	Hospital capacity Medium	1.120	0.907	1.382	0.292
	Hospital capacity Small	0.270	0.202	0.363	0.000

Table A.4: Estimated odds ratios for covariate effects on the probability of moving from ICU to death. The probability of moving from ICU to discharge is the complement, 1 minus the probability of death.

Predicted vs observed probabilities of moving from ICU to either post-ICU or death are displayed in Figures A.6 to A.9, for selected covariates. The probability of death from ICU increases with age, is higher for men than women, is highest for patients admitted in March, is higher for patients with comorbidities and care home residents, and is lower for healthcare workers and the smallest hospitals (many of which have no ICU beds).

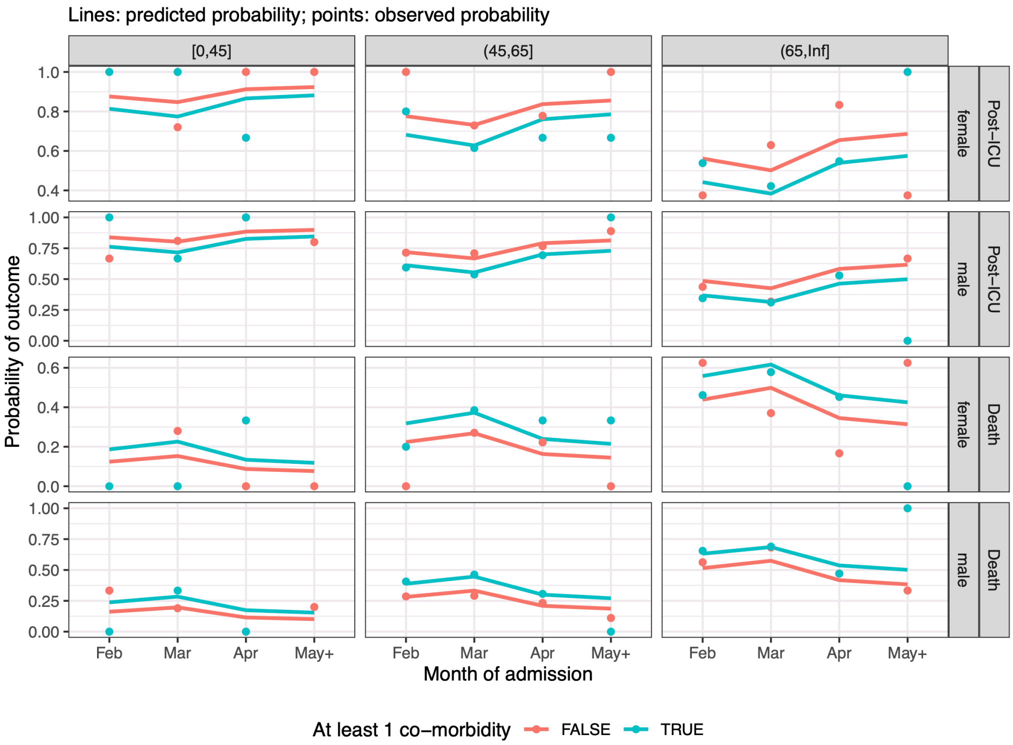

Figure A.6: Predicted vs observed probabilities of moving from ICU to either a post-ICU stay or death, by age, sex, month of admission and co-morbidity. All other covariates are set to their baseline values, i.e. these probabilities are for patients who are not care home residents nor healthcare workers, and who are in large hospitals.

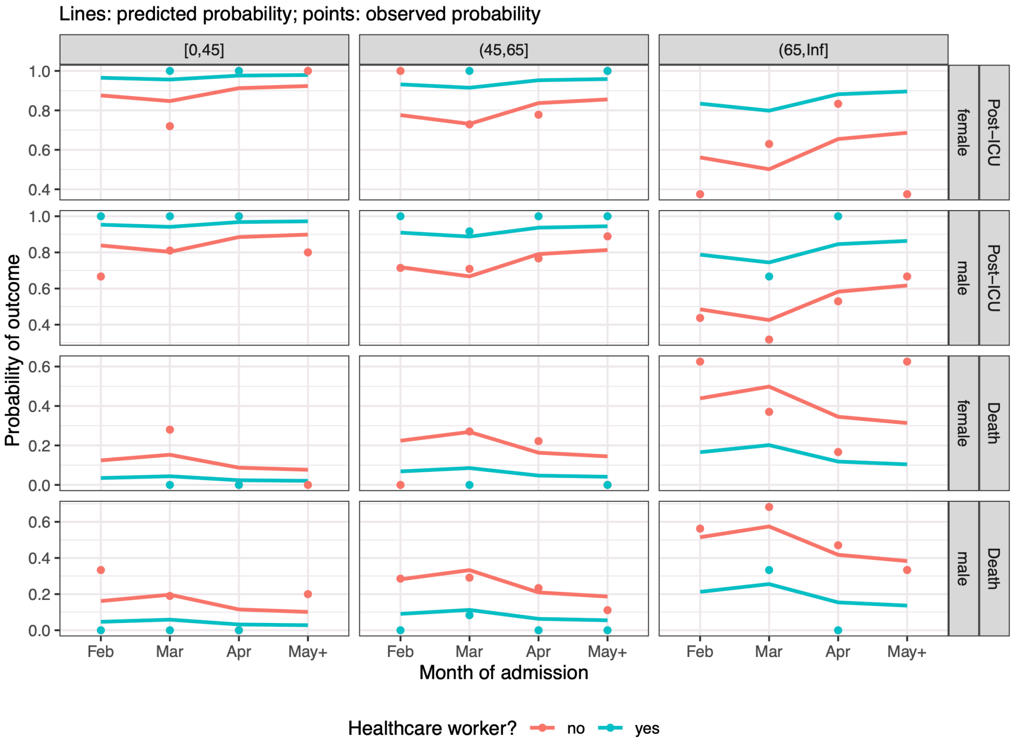

Figure A.7: Predicted vs observed probabilities of moving from ICU to either a post-ICU stay or death, by age, sex, month of admission and healthcare worker status. All other covariates are set to their baseline values, i.e. these probabilities are for patients who have no co-morbidity, are not care home residents and who are in large hospitals.

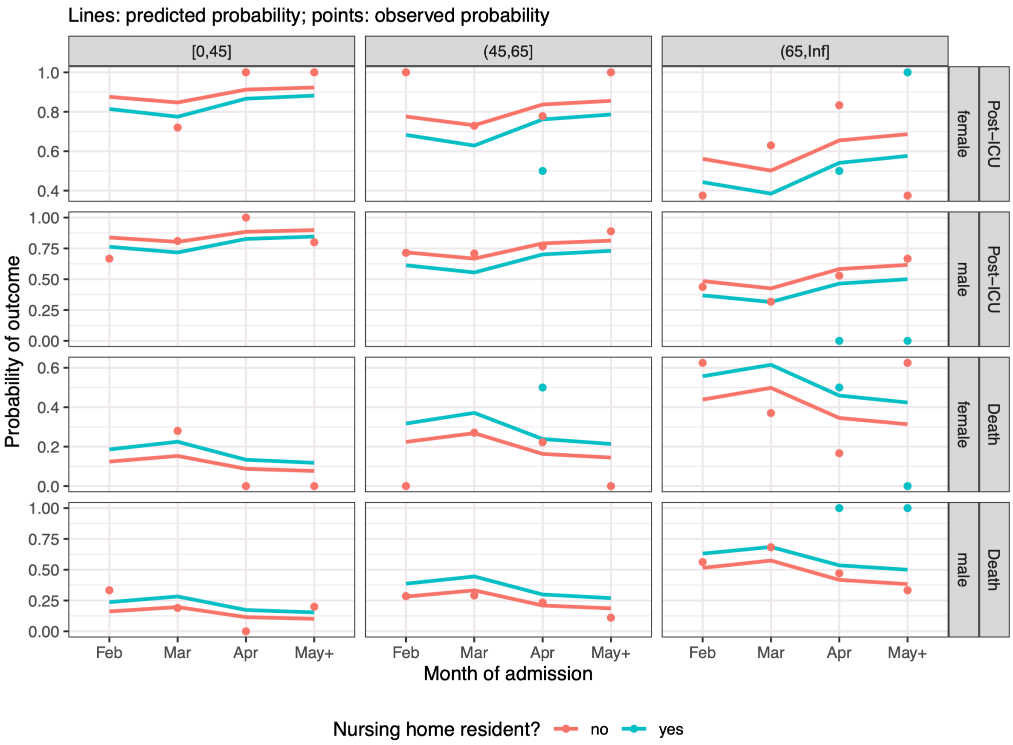

Figure A.8: Predicted vs observed probabilities of moving from ICU to either a post-ICU stay or death, by age, sex, month of admission and care home residence. All other covariates are set to their baseline values, i.e. these probabilities are for patients who have no co-morbidity, are not healthcare workers and who are in large hospitals.

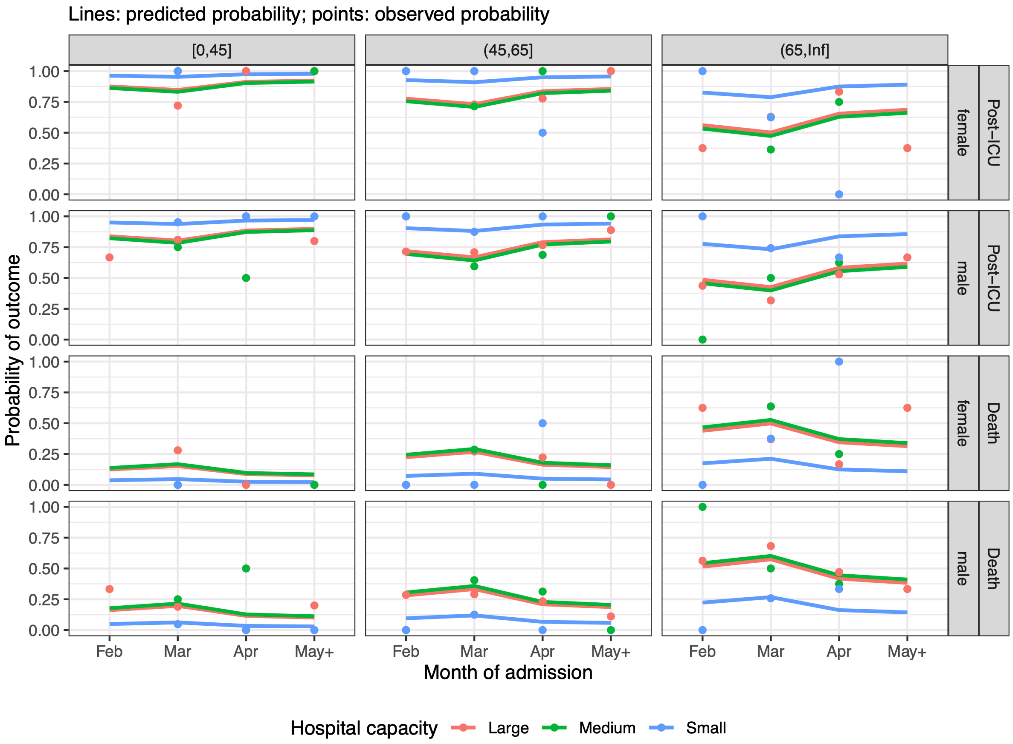

Figure A.9: Predicted vs observed probabilities of moving from ICU to either a post-ICU stay or death, by age, sex, month of admission and hospital bed capacity. All other covariates are set to their baseline values, i.e. these probabilities are for patients who have no co-morbidity and are not healthcare workers nor care home residents.

A.2.3 Post-ICU stay to discharge or death

Due to small sample sizes for transitions from a post-ICU stay, the two youngest age groups are combined into a single covariate level [0,65]; and admissions in May and June are combined into a single covariate level. As there are so few care home residents in the post-ICU state, care home residency is not considered as a covariate for transitions from the post-ICU state. For the transition from a post-ICU stay to death, the selected binomial logistic regression model includes only main effects for age, sex, month of admission, and whether the patient has at least one co-morbidity (Table A.5). Age group is the only strongly significant covariate with a large effect giving lower probability of death to those aged under 65. Calendar month of admission has large effects compared to March, but the significance of the differences and pattern over time is not clear (Figure A.10).

Transition to	Covariate level	OR at baseline	95% CI		p
Death	Age group [0,65]	0.273	0.195	0.383	0.000
	Sex male	0.936	0.646	1.355	0.725
	Month of admission Feb	3.541	2.164	5.793	0.000
	Month of admission Apr	0.860	0.540	1.370	0.526
	Month of admission May-Jun	2.056	0.752	5.627	0.160
	At least 1 co-morbidity	1.392	0.983	1.970	0.062

Table A.5: Estimated odds ratios for covariate effects on the probability of moving from a post-ICU stay to death. The probability of moving from a post-ICU stay to discharge is the complement, 1 minus the probability of death.

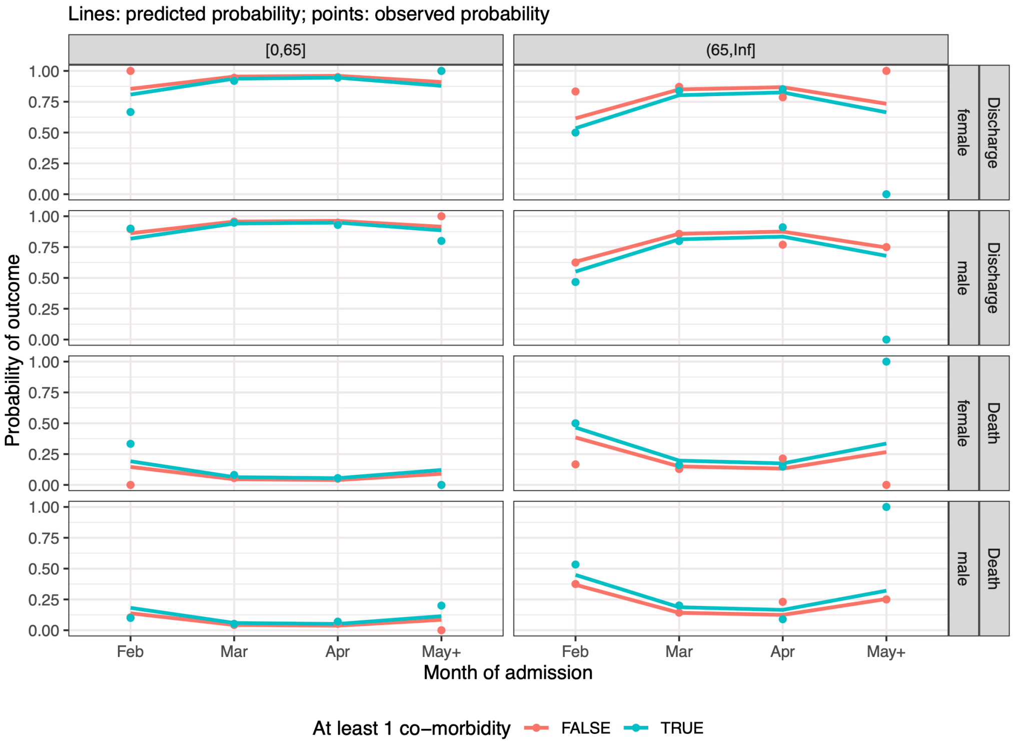

Figure A.10: Predicted vs observed probabilities of moving from a post-ICU stay to discharge or death, by age, sex, month of admission and co-morbidity.

A.3 Multivariable models - times to next events

The selected models for each transition are summarised in Table A.6.

Transition time	Distribution	Selected covariates	Log-likelihood	AIC
Hospital to discharge	generalised gamma	age group * (sex + month of admission + co-morbidity + care home resident + healthcare worker + hospital bed capacity)	-102,346	204,761
Hospital to ICU admission	generalised gamma	age group + sex + month of admission	-9,697	19,415
Hospital to death	generalised gamma	age group + sex + month of admission + co-morbidity + care home resident + healthcare worker + hospital bed capacity	-29,615	59,257
ICU to post-ICU	generalised gamma	age group + sex + month of admission + co-morbidity + care home resident	-8,059	16,141
ICU to death	gamma	age group + sex + month of admission + co-morbidity + care home resident	-5,430	10,880
Post-ICU to discharge	gamma	age group + sex + month of admission + co-morbidity + healthcare worker + hospital bed capacity	-6,410	12,843
Post-ICU to death	log-normal	healthcare worker	-604	1,214

Table A.6: Summary of selected covariates and distributions for each time to a next event. The star (*) notation denotes both main effects and interactions.

A.3.1 Covariate effects on times to next events

The covariate effects on the distributions of times to next events are expressed as multiplicative factors ("expected time ratios", ETR) that lengthen or shorten the times. An effect of size x implies that the expected time is x times longer or shorter than the baseline time. Tables A.7 to A.13 display the covariate effects on each time to a next event. The corresponding estimated distributions of the times to a next event are shown in Section A.3.2.

Covariate level	ETR	95% CI		p-value
Age group (45,65]	0.531	0.497	0.568	0.000
Age group [0,45]	0.411	0.378	0.446	0.000
Sex male	0.945	0.911	0.980	0.001

Month of admission Feb	1.663	1.526	1.812	0.000
Month of admission Apr	1.017	0.973	1.064	0.223
Month of admission May	0.763	0.698	0.833	0.000
Month of admission Jun	0.488	0.421	0.567	0.000
At least 1 co-morbidity	1.120	1.071	1.172	0.000
Care home resident	1.015	0.934	1.103	0.365
Healthcare worker	0.563	0.422	0.749	0.000
Hospital capacity Medium	1.151	1.091	1.215	0.000
Hospital capacity Small	1.353	1.294	1.414	0.000
Age group (45,65] : sex male	1.271	1.203	1.343	0.000
Age group [0,45] : sex male	1.214	1.125	1.309	0.000
Age group (45,65] : Month of admission Feb	1.178	1.008	1.377	0.020
Age group [0,45] : Month of admission Feb	1.043	0.844	1.290	0.348
Age group (45,65] : Month of admission Apr	0.929	0.870	0.992	0.014
Age group [0,45] : Month of admission Apr	0.918	0.842	1.001	0.026
Age group (45,65] : Month of admission May	0.864	0.747	1.000	0.025
Age group [0,45] : Month of admission May	0.902	0.778	1.045	0.085
Age group (45,65] : Month of admission Jun	1.124	0.865	1.461	0.190
Age group [0,45] : Month of admission Jun	1.304	1.027	1.657	0.015
Age group (45,65] : At least 1 co-morbidity	1.052	0.990	1.117	0.050
Age group [0,45] : At least 1 co-morbidity	1.096	0.985	1.220	0.046
Age group (45,65] : Care home resident	1.992	1.526	2.599	0.000
Age group [0,45] : Care home resident	1.601	0.944	2.716	0.040
Age group (45,65] : Healthcare worker	1.240	0.924	1.664	0.076
Age group [0,45] : Healthcare worker	1.154	0.850	1.565	0.179
Age group (45,65] : Hospital capacity Medium	0.908	0.836	0.985	0.010
Age group [0,45] : Hospital capacity Medium	0.818	0.732	0.915	0.000
Age group (45,65] : Hospital capacity Small	0.963	0.896	1.035	0.154
Age group [0,45] : Hospital capacity Small	1.057	0.939	1.188	0.180

Table A.7: Estimated covariate effects on time from admission to a hospital ward to discharge without an ICU stay, conditional on being discharged. Note that the main effects are at the baseline levels of all other covariates. The effects at other levels are obtained as functions of the main effects and the interaction effects.

Covariate level	ETR	95% CI		p-value
Age group (45,65]	0.812	0.747	0.883	0.000
Age group [0,45]	0.648	0.549	0.766	0.000
Sex male	0.915	0.830	1.009	0.038
Month of admission Feb	1.052	0.890	1.244	0.275
Month of admission Apr	0.826	0.734	0.930	0.001
Month of admission May	0.614	0.428	0.879	0.004
Month of admission Jun	0.294	0.146	0.593	0.000

Table A.8: Estimated covariate effects on time from admission to a hospital ward to an ICU admission, conditional on being admitted to ICU.

Covariate level	ETR	95% CI		p-value
Age group [0,65]	1.050	0.961	1.146	0.140
Sex male	0.913	0.875	0.954	0.000
Month of admission Feb	3.185	2.880	3.522	0.000
Month of admission Apr	1.269	1.198	1.343	0.000

Month of admission May	1.257	1.087	1.453	0.001
Month of admission Jun	0.781	0.542	1.124	0.091
At least 1 co-morbidity	0.975	0.917	1.036	0.204
Care home resident	0.796	0.736	0.860	0.000
Healthcare worker	0.642	0.287	1.437	0.141
Hospital capacity Medium	1.061	1.000	1.125	0.024
Hospital capacity Small	1.144	1.078	1.215	0.000

Table A.9: Estimated covariate effects on time from admission to a hospital ward to death, conditional on dying.

Covariate level	ETR	95% CI		p-value
Age group (45,65]	0.896	0.820	0.980	0.008
Age group [0,45]	0.716	0.618	0.830	0.000
Sex male	1.086	0.991	1.191	0.039
Month of admission Feb	0.797	0.674	0.941	0.004
Month of admission Apr	0.833	0.747	0.929	0.001
Month of admission May+Jun	0.620	0.469	0.819	0.000
At least 1 co-morbidity	1.028	0.949	1.115	0.249
Care home resident	0.480	0.262	0.878	0.009

Table A.10: Estimated covariate effects on time from ICU admission to a post-ICU stay, conditional on experiencing a post-ICU stay.

Covariate level	ETR	95% CI		p-value
Age group (45,65]	1.063	0.977	1.155	0.077
Age group [0,45]	1.148	0.888	1.486	0.146
Sex male	0.941	0.847	1.044	0.125
Month of admission Feb	1.148	0.976	1.351	0.048
Month of admission Apr	1.191	1.039	1.365	0.006
Month of admission May+Jun	0.377	0.248	0.572	0.000
At least 1 co-morbidity	0.915	0.837	1.000	0.026
Care home resident	0.596	0.334	1.064	0.040

Table A.11: Estimated covariate effects on time from ICU admission to death, conditional on dying in ICU.

Covariate level	ETR	95% CI		p-value
Age group [0,65]	0.831	0.769	0.897	0.000
sex male	0.937	0.866	1.013	0.051
Month of admission Feb	0.998	0.846	1.177	0.489
Month of admission Apr	0.835	0.762	0.915	0.000
Month of admission May+Jun	0.608	0.472	0.783	0.000
At least 1 co-morbidity	1.063	0.995	1.137	0.036
Healthcare worker	0.818	0.702	0.953	0.005
Hospital capacity Medium	1.179	1.063	1.307	0.001
Hospital capacity Small	1.203	1.090	1.328	0.000

Table A.12: Estimated covariate effects on time from a post-ICU stay to discharge, conditional on being discharged.

Covariate level	ETR	95% CI		p-value
Healthcare worker	0.383	0.168	0.870	0.011

Table A.13: Estimated covariate effects on time from a post-ICU stay to death, conditional on dying while on a post-ICU ward.

A.3.2 Estimated distributions of times to next events

The estimated median (95% confidence interval for the median) and inter-quartile range (25%-ile and 75%-ile) of the time-to-next-event distributions are shown for selected covariate combinations in Figures A.11 to A.27. The confidence interval for the median (solid lines) reflects uncertainty in the estimate, whereas the inter-quartile range of the distribution (dashed lines) represents heterogeneity in the times to next event within the displayed covariate stratum.

A.3.2.1 Hospital admission to discharge without ICU admission

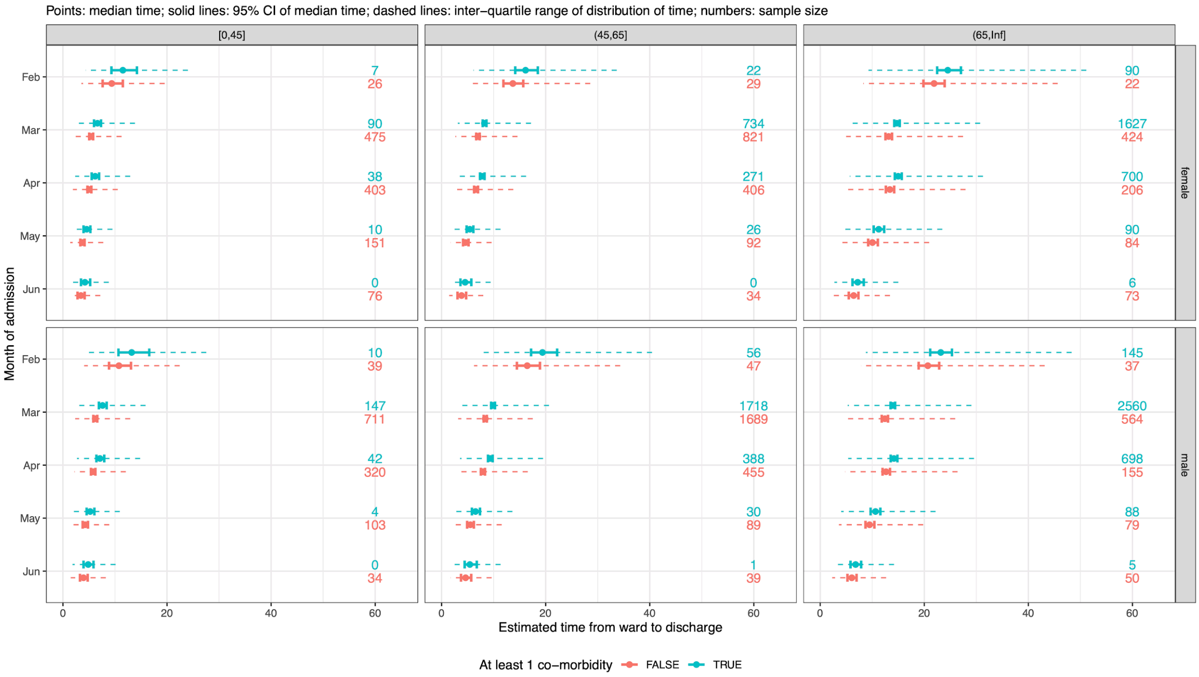

Figure A.11: Median (95% CI of median, point and solid line) and inter-quartile range (dashed line) of the distribution of time from admission to a hospital ward to discharge without ICU admission, conditional on being discharged, by age, sex, month of admission and co-morbidity. All other covariates are set to their baseline levels, i.e. these estimates are for patients who are neither healthcare workers nor care home residents, and who are in large hospitals. The numbers represent the sample size in each stratum.

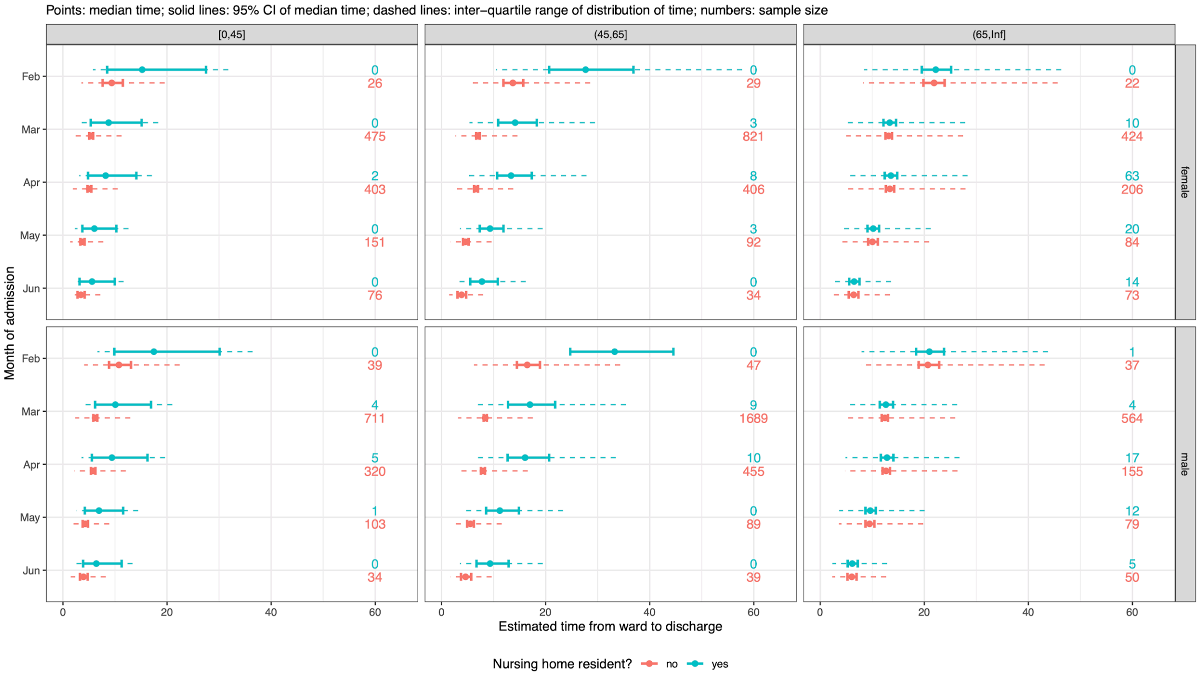

Figure A.12: Median (95% CI of median, point and solid line) and inter-quartile range (dashed line) of the distribution of time from admission to a hospital ward to discharge without ICU admission, conditional on being discharged, by age, sex, month of admission and care home residency. All other covariates are set to their baseline levels, i.e. these estimates are for

patients with no co-morbidity, who are not healthcare workers, and who are in large hospitals. The numbers represent the sample size in each stratum.

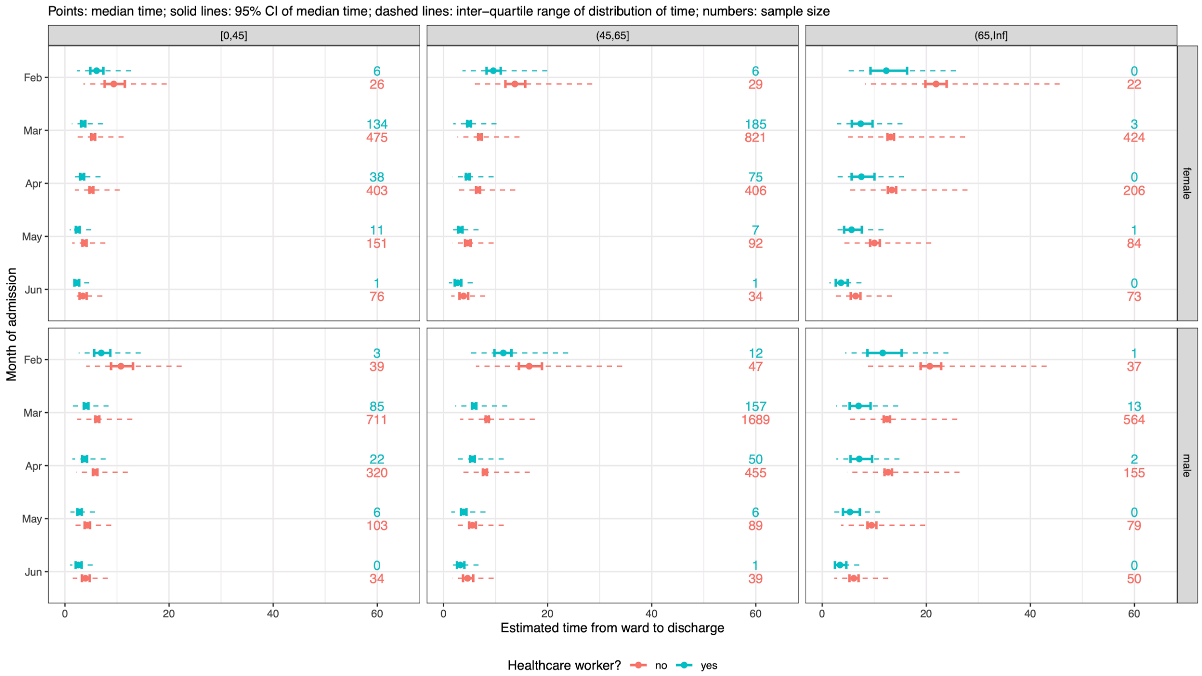

Figure A.13: Median (95% CI of median, point and solid line) and inter-quartile range (dashed line) of the distribution of time from admission to a hospital ward to discharge without ICU admission, conditional on being discharged, by age, sex, month of admission and healthcare worker status. All other covariates are set to their baseline levels, i.e. these estimates are for patients with no co-morbidity, who are not care home residents, and who are in large hospitals. The numbers represent the sample size in each stratum.

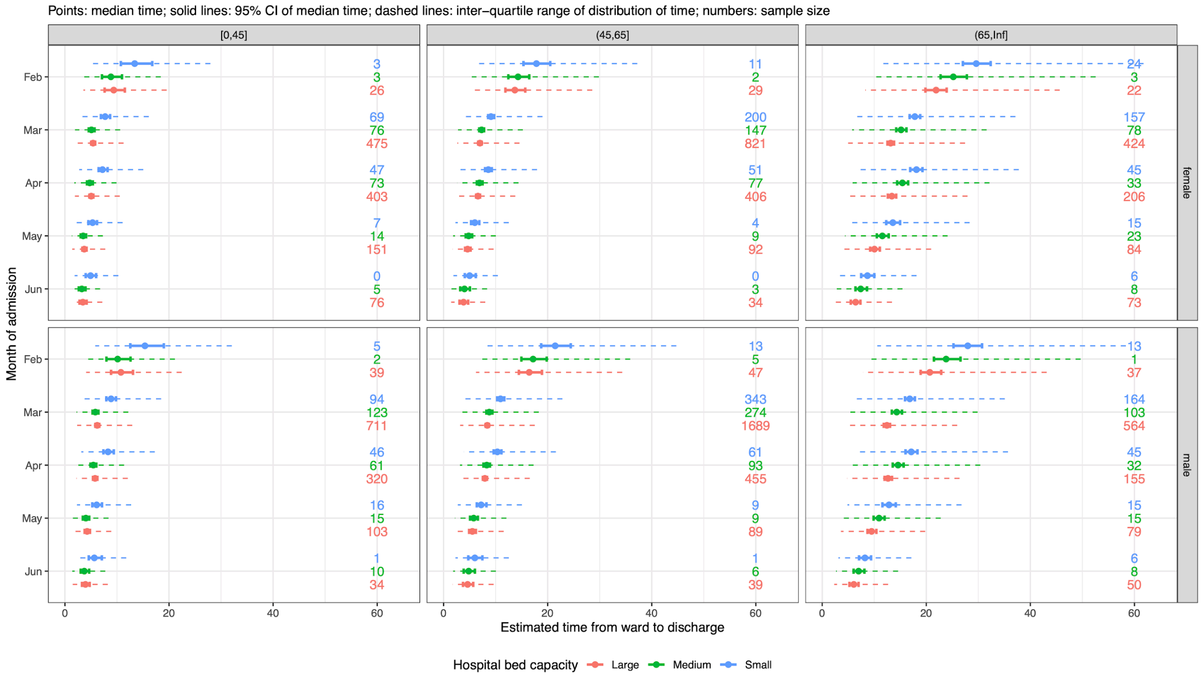

Figure A.14: Median (95% CI of median, point and solid line) and inter-quartile range (dashed line) of the distribution of time from admission to a hospital ward to discharge without ICU admission, conditional on being discharged, by age, sex, month of admission and hospital bed capacity. All other covariates are set to their baseline levels, i.e. these estimates are for patients with no co-morbidity, who are neither care home residents nor healthcare workers. The numbers represent the sample size in each stratum.

A.3.2.2 Hospital admission to ICU admission

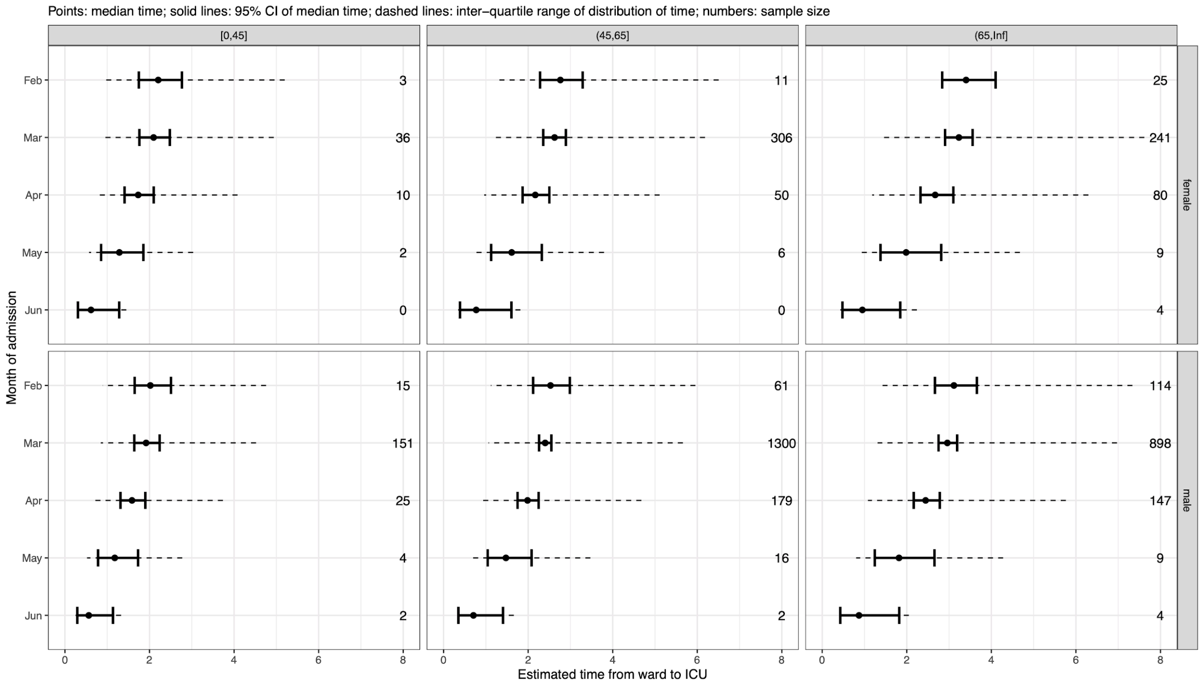

Figure A.15: Median (95% CI of median, point and solid line) and inter-quartile range (dashed line) of the distribution of time from admission to a hospital ward to ICU admission, conditional on being admitted to ICU, by age, sex, and month of admission. The numbers represent the sample size in each stratum.

A.3.2.3 Hospital admission to death without ICU admission

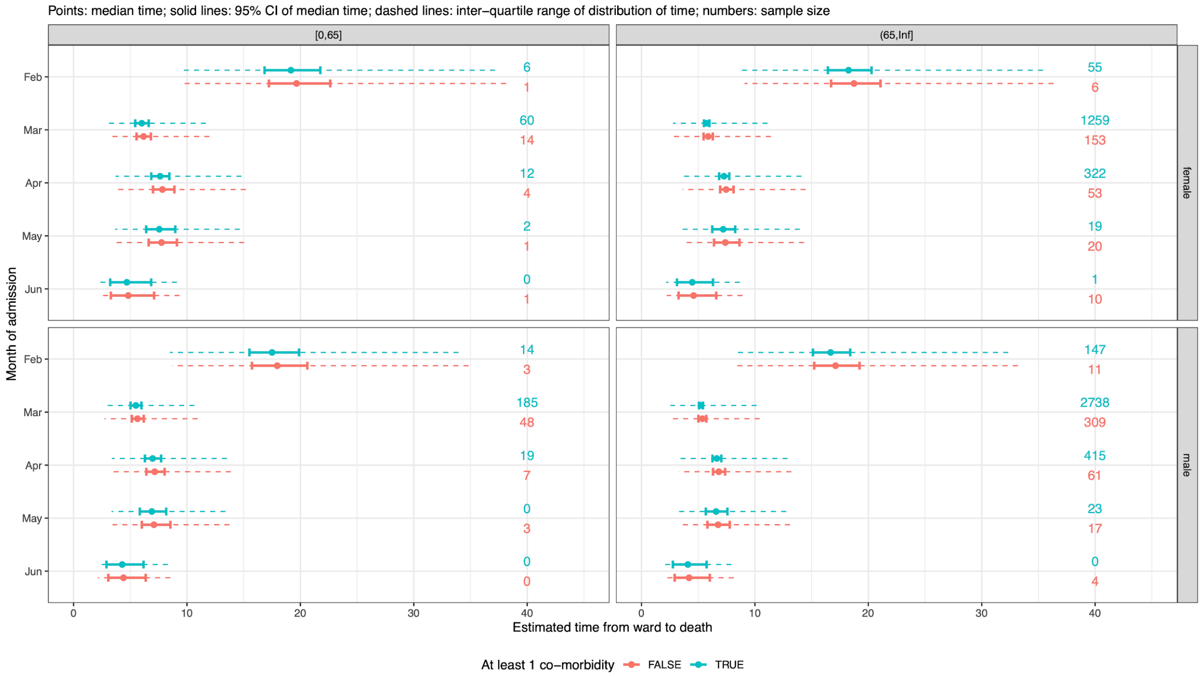

Figure A.16: Median (95% CI of median, point and solid line) and inter-quartile range (dashed line) of the distribution of time from admission to a hospital ward to death without ICU admission, conditional on dying, by age, sex, month of admission and co-morbidity. All other covariates are set to their baseline levels, i.e. these estimates are for patients who are neither care home residents nor healthcare workers, and who are in large hospitals. The numbers represent the sample size in each stratum.

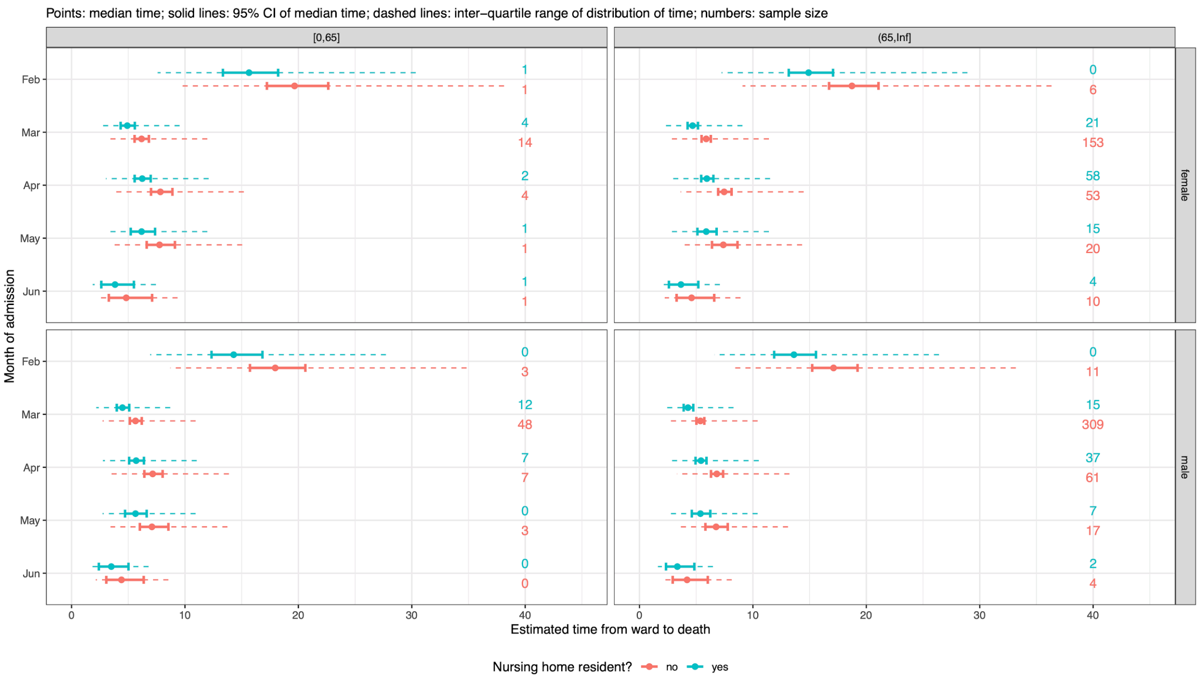

Figure A.17: Median (95% CI of median, point and solid line) and inter-quartile range (dashed line) of the distribution of time from admission to a hospital ward to death without ICU admission, conditional on dying, by age, sex, month of admission and care home residency. All other covariates are set to their baseline levels, i.e. these estimates are for patients who have no co-morbidity, are not healthcare workers, and who are in large hospitals. The numbers represent the sample size in each stratum.

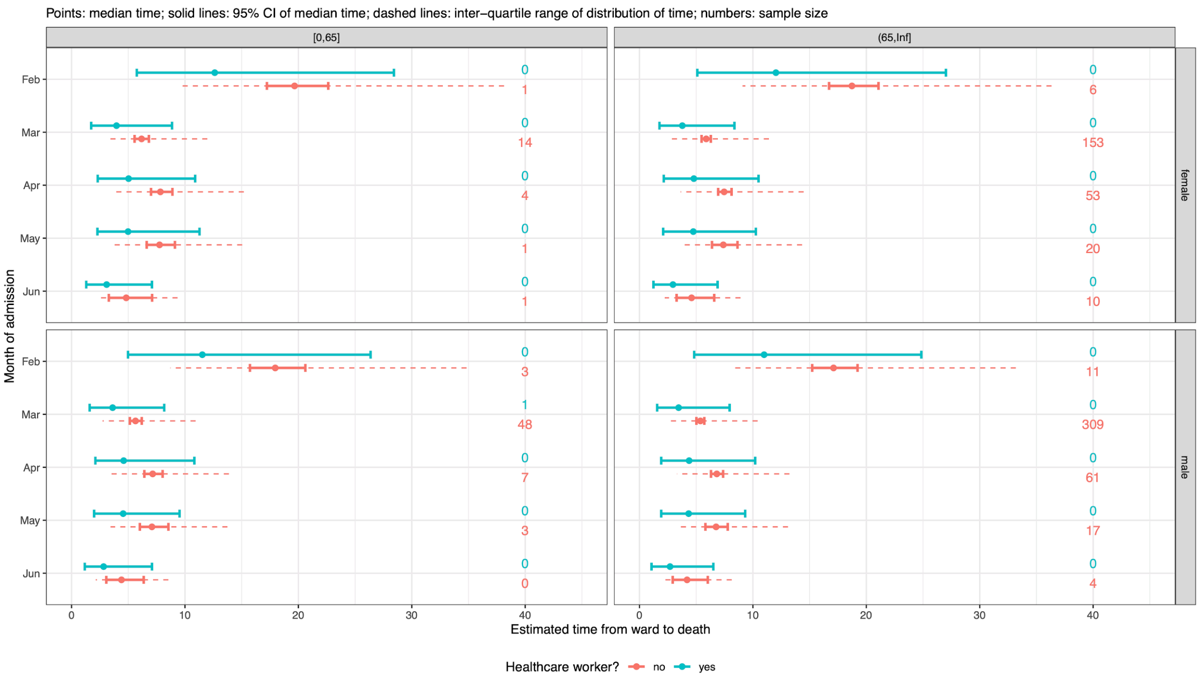

Figure A.18: Median (95% CI of median, point and solid line) and inter-quartile range (dashed line) of the distribution of time from admission to a hospital ward to death without ICU admission, conditional on dying, by age, sex, month of admission and healthcare worker status. All other covariates are set to their baseline levels, i.e. these estimates are for patients who have no co-morbidity, are not care home residents, and who are in large hospitals. The numbers represent the sample size in each stratum.

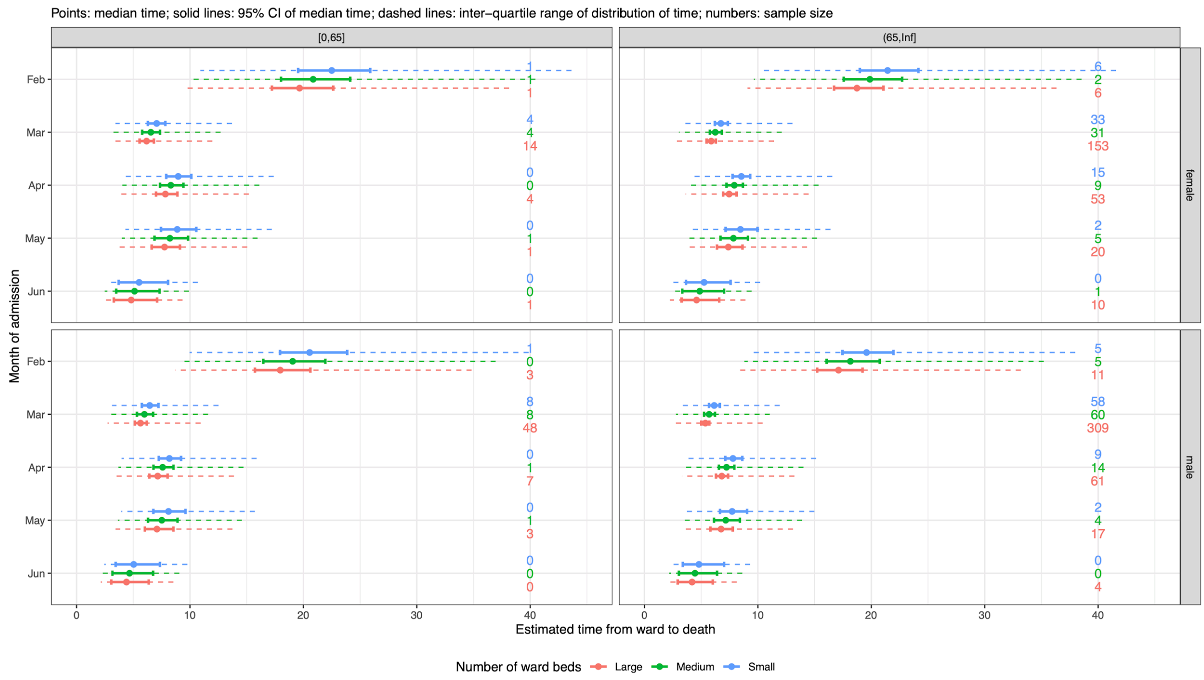

Figure A.19: Median (95% CI of median, point and solid line) and inter-quartile range (dashed line) of the distribution of time from admission to a hospital ward to death without ICU admission, conditional on dying, by age, sex, month of admission and hospital bed capacity. All other covariates are set to their baseline levels, i.e. these estimates are for patients who have no co-morbidity and are neither healthcare workers nor care home residents. The numbers represent the sample size in each stratum.

A.3.2.4 ICU admission to post-ICU stay

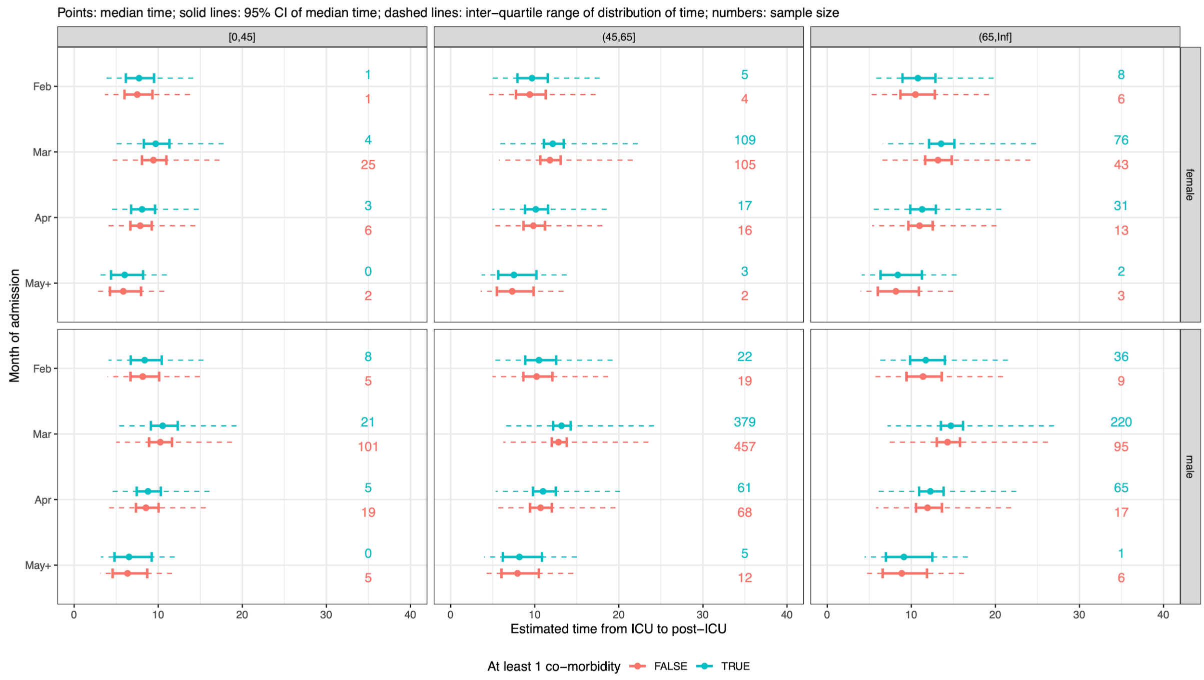

Figure A.20: Median (95% CI of median, point and solid line) and inter-quartile range (dashed line) of the distribution of time from ICU admission to a post-ICU stay, conditional on a post-ICU stay, by age, sex, month of admission and co-morbidity. All other covariates are set to their baseline levels, i.e. these estimates are for patients who are not care home residents. The numbers represent the sample size in each stratum.

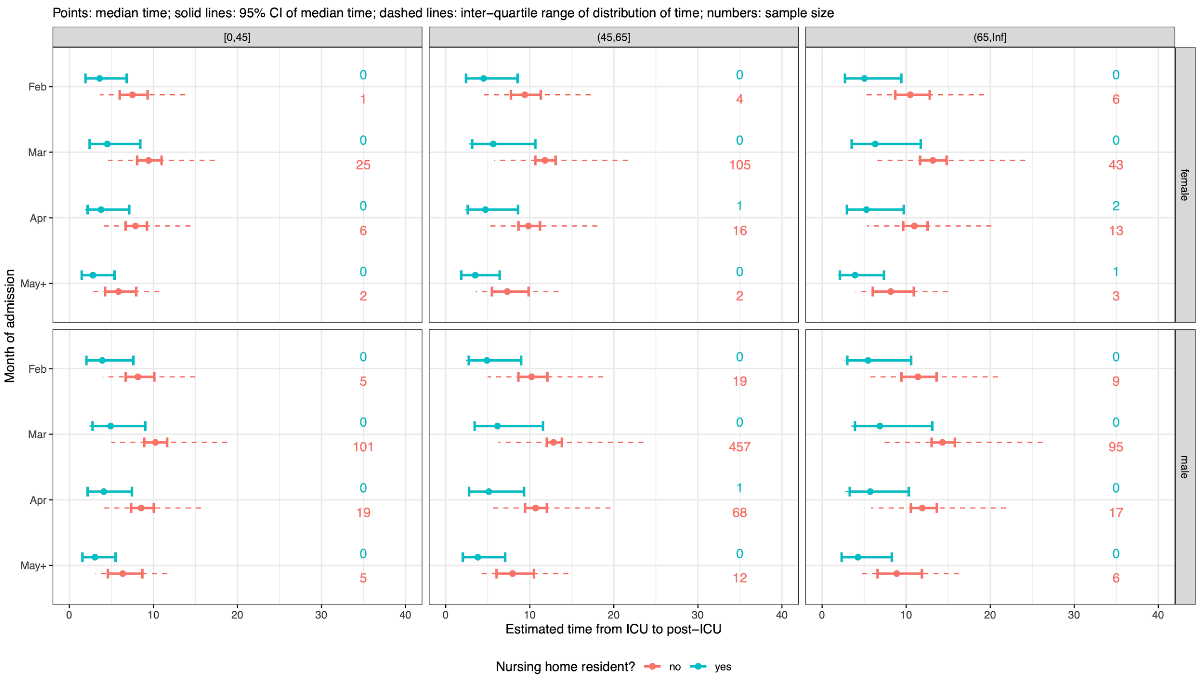

Figure A.21: Median (95% CI of median, point and solid line) and inter-quartile range (dashed line) of the distribution of time from ICU admission to a post-ICU stay, conditional on a post-ICU stay, by age, sex, month of admission and care home residence. All other covariates are set to their baseline levels, i.e. these estimates are for patients who have no co-morbidity. The numbers represent the sample size in each stratum.

A.3.2.5 ICU admission to death

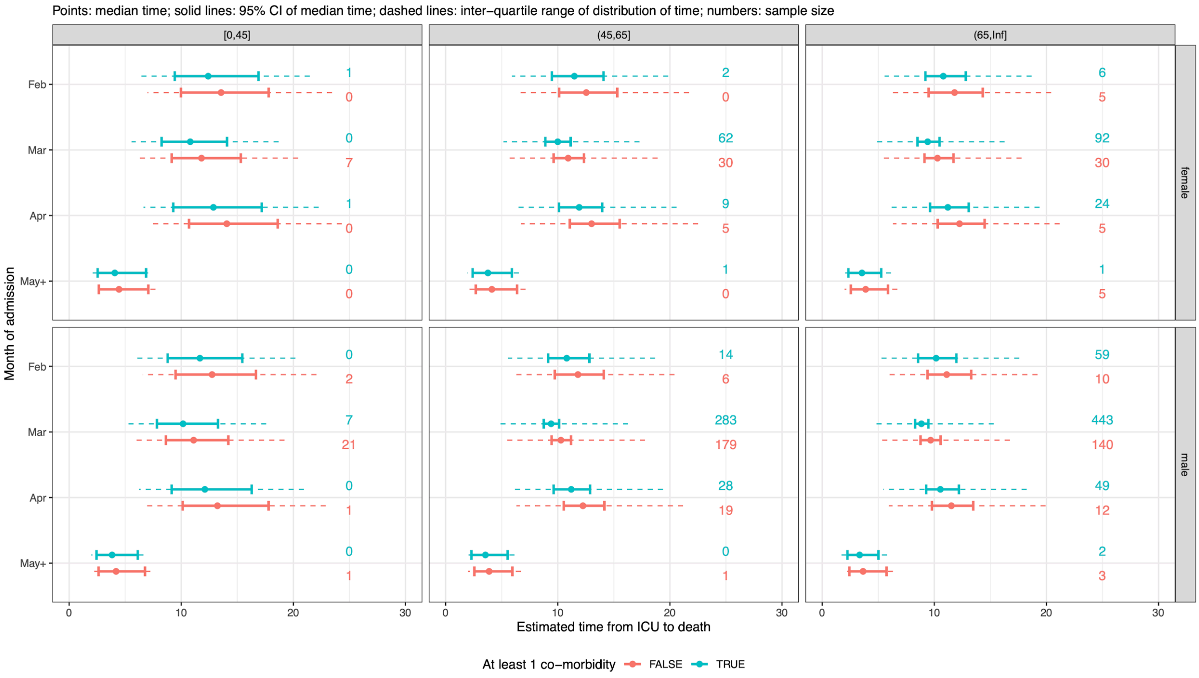

Figure A.22: Median (95% CI of median, point and solid line) and inter-quartile range (dashed line) of the distribution of time from ICU admission to death, conditional on dying in ICU, by age, sex, month of admission and co-morbidity. All other covariates are set to their baseline levels, i.e. these estimates are for patients who are not care home residents. The numbers represent the sample size in each stratum.

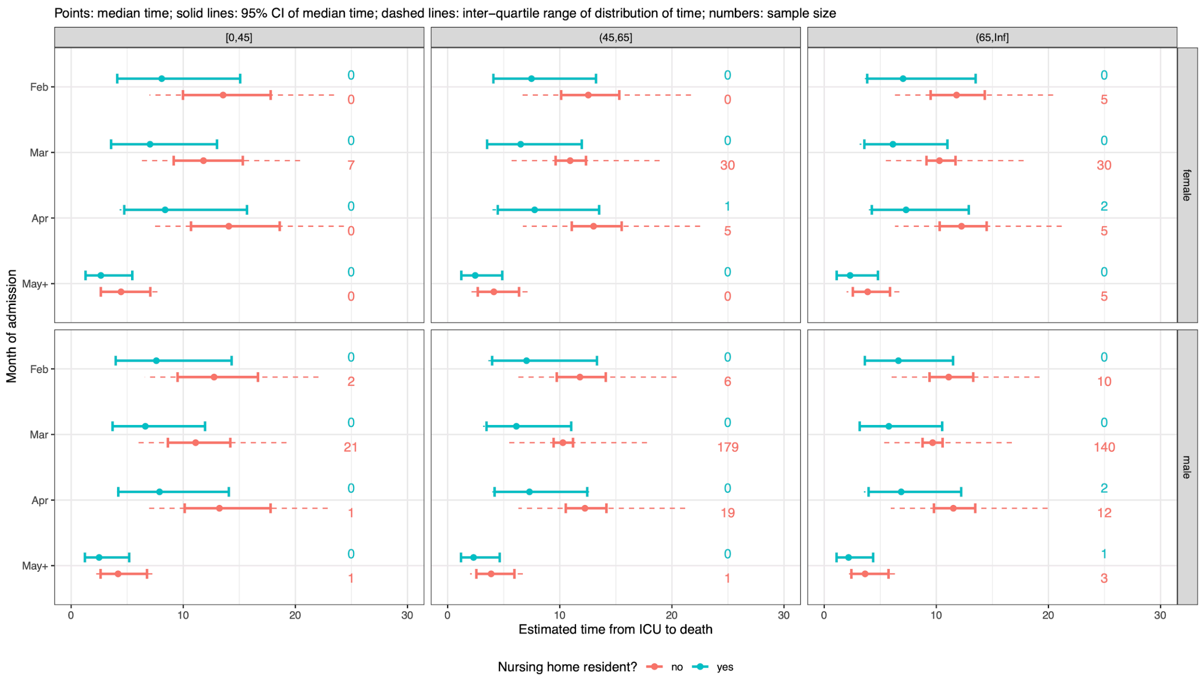

Figure A.23: Median (95% CI of median, point and solid line) and inter-quartile range (dashed line) of the distribution of time from ICU admission to death, conditional on dying in ICU, by age, sex, month of admission and care home residence. All other covariates are set to their baseline levels, i.e. these estimates are for patients who have no co-morbidity. The numbers represent the sample size in each stratum.

A.3.2.6 Post-ICU stay to discharge

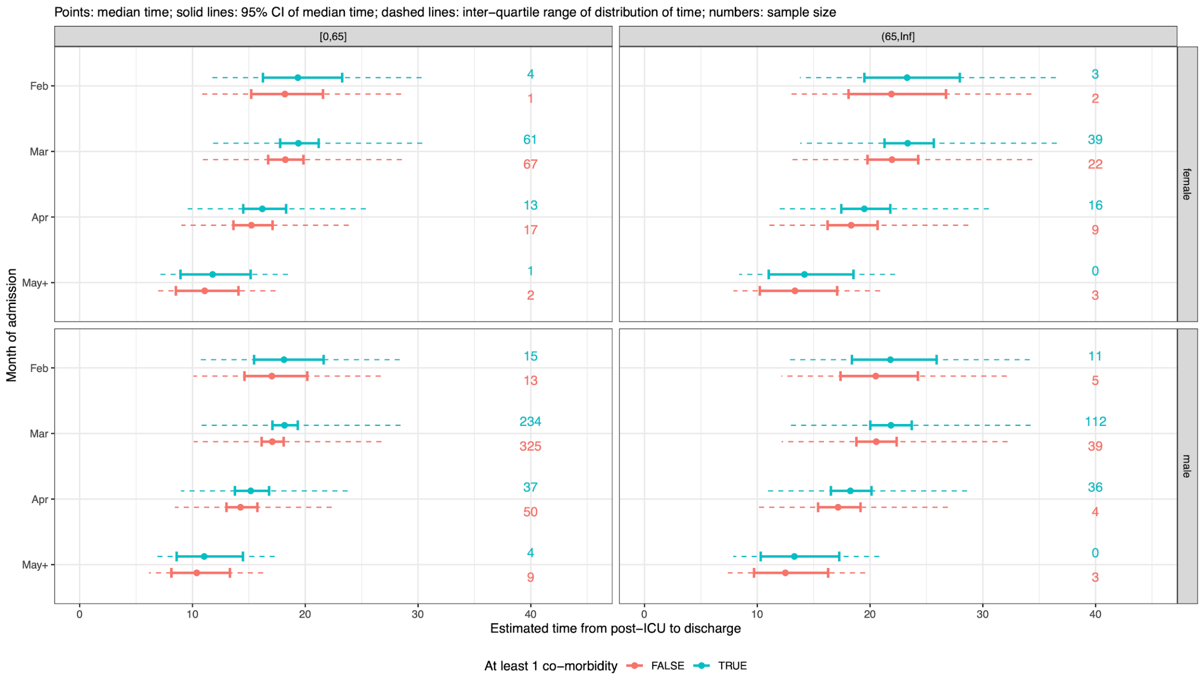

Figure A.24: Median (95% CI of median, point and solid line) and inter-quartile range (dashed line) of the distribution of time from a post-ICU stay to discharge, conditional on being discharged, by age, sex, month of admission and co-morbidity. All other covariates are set to their baseline levels, i.e. these estimates are for patients who are not healthcare workers and who are in large hospitals. The numbers represent the sample size in each stratum.

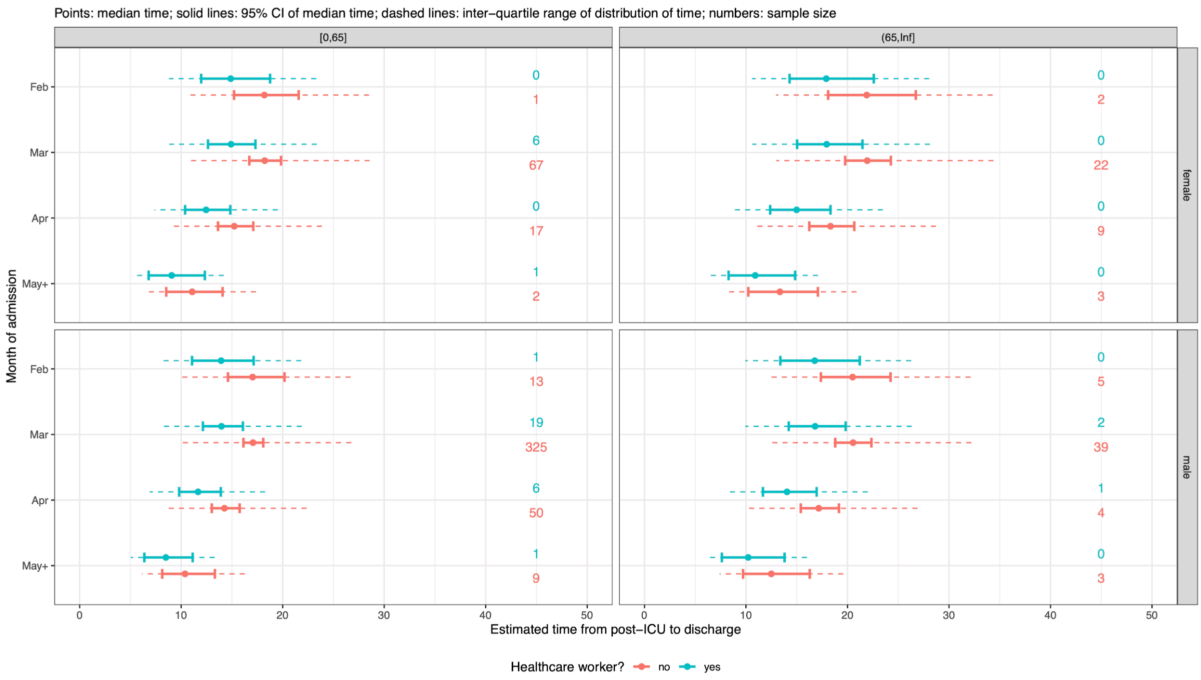

Figure A.25: Median (95% CI of median, point and solid line) and inter-quartile range (dashed line) of the distribution of time from a post-ICU stay to discharge, conditional on being discharged, by age, sex, month of admission and healthcare worker status. All other covariates are set to their baseline levels, i.e. these estimates are for patients who have no co-morbidity and who are in large hospitals. The numbers represent the sample size in each stratum.

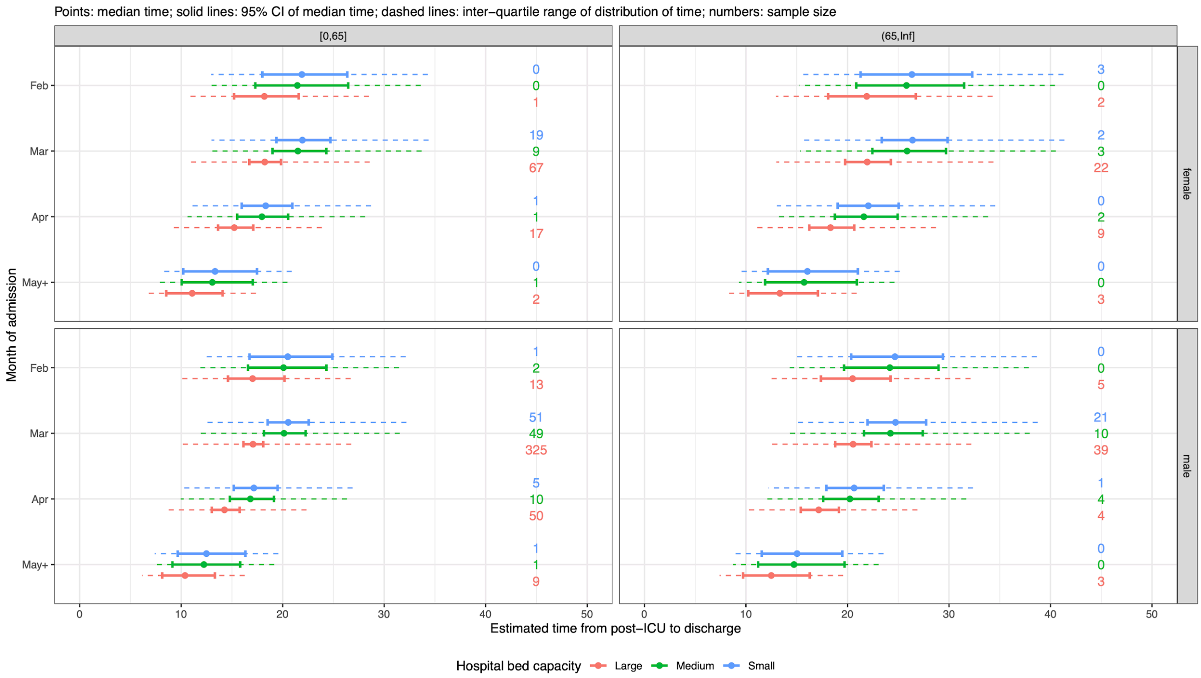

Figure A.26: Median (95% CI of median, point and solid line) and inter-quartile range (dashed line) of the distribution of time from a post-ICU stay to discharge, conditional on being discharged, by age, sex, month of admission and hospital bed capacity. All other covariates are set to their baseline levels, i.e. these estimates are for patients who have no co-morbidity and who are not healthcare workers. The numbers represent the sample size in each stratum.

A.3.2.7 Post-ICU stay to death

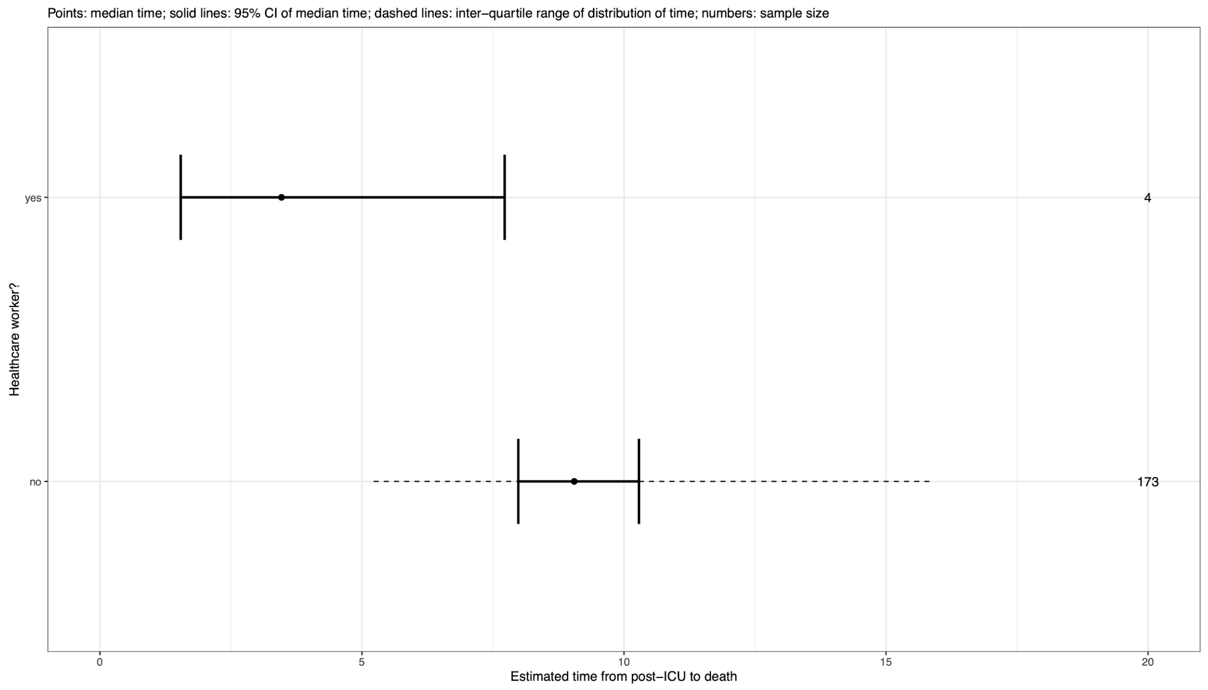

Figure A.27: Median (95% CI of median, point and solid line) and inter-quartile range (dashed line) of the distribution of time from a post-ICU stay to death, conditional on dying in a post-ICU ward, by healthcare worker status.

A.3.3 Goodness-of-fit

We assess the goodness-of-fit of the transition-specific time-to-event models by comparing the parametric cumulative incidence functions for each next event with Aalen-Johansen non-parametric estimates, for selected covariate combinations. In each of Figures A.28 to A.30, for each age, sex and month of admission stratum, these comparisons are made with all other covariates in each model set to their baseline values, if the covariate appears in the model (month of admission March, no co-morbidities, not a care home resident, not a healthcare worker, large hospital bed capacity). The goodness-of-fit is generally good, with only some slight lack of fit in strata with small sample sizes.

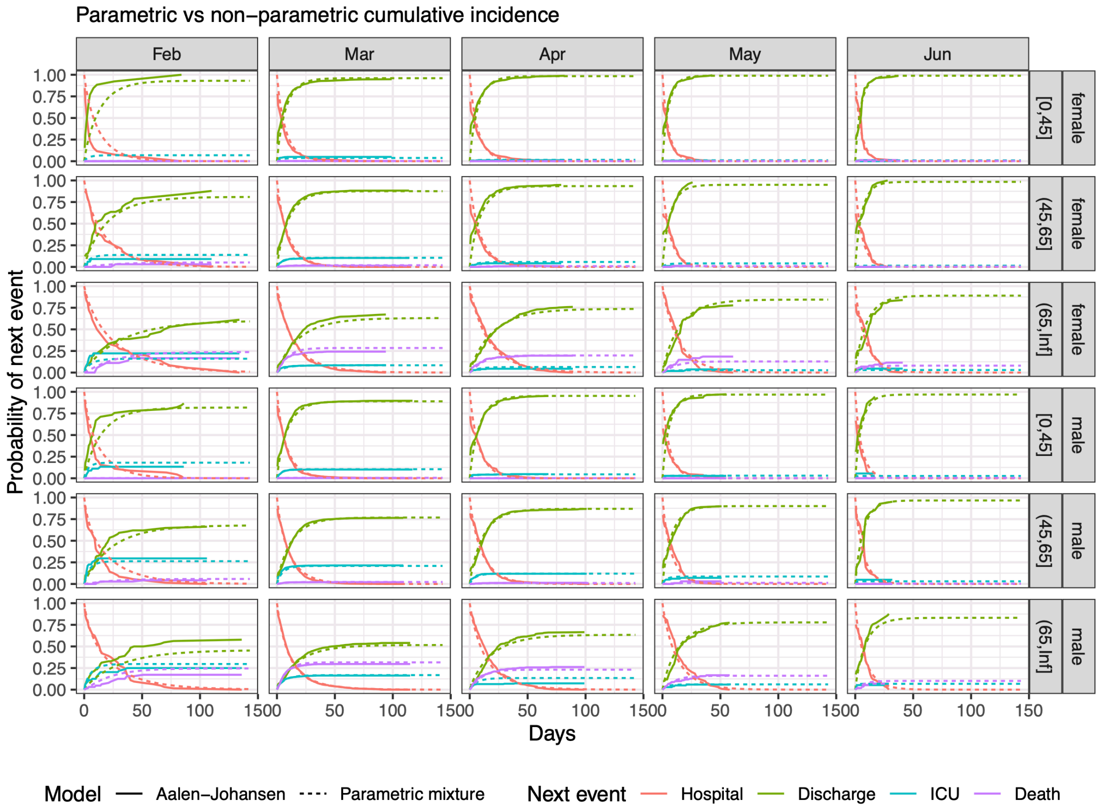

Figure A.28: Cumulative incidence curves for next events from Hospital admission, by age, sex, and month of admission.

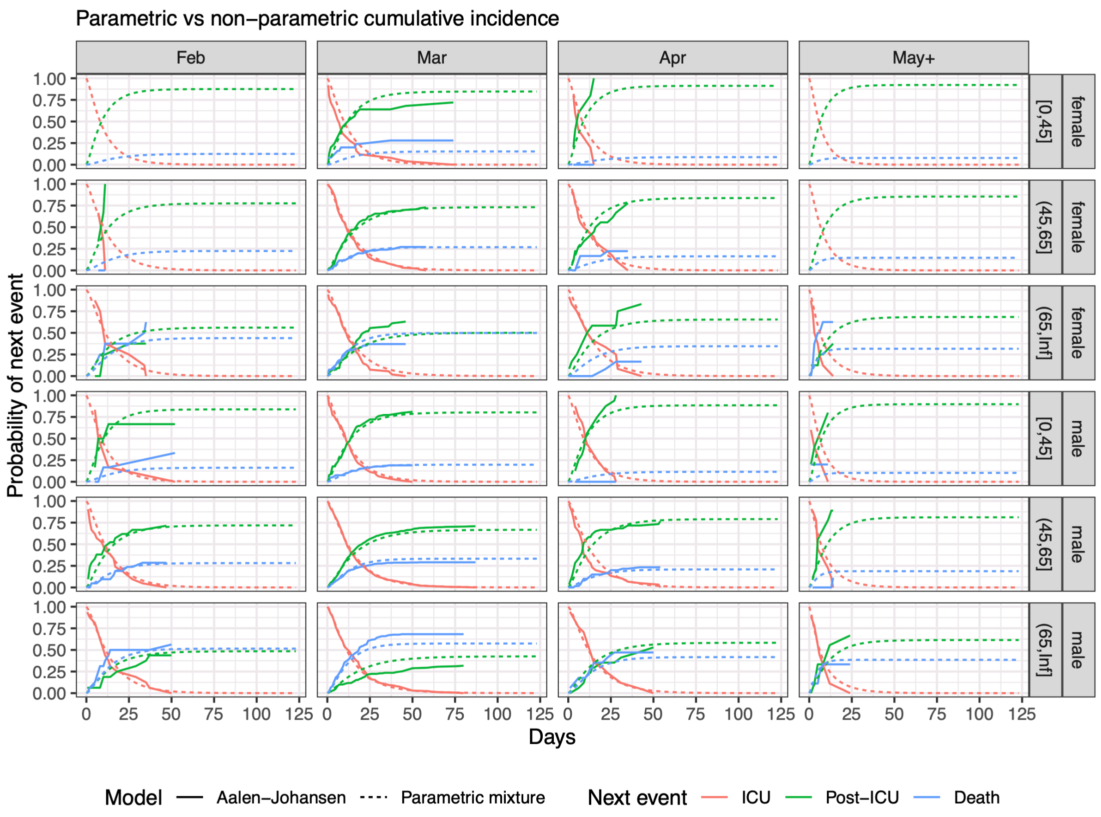

Figure A.29: Cumulative incidence curves for next events from ICU admission, by age, sex, and month of admission.

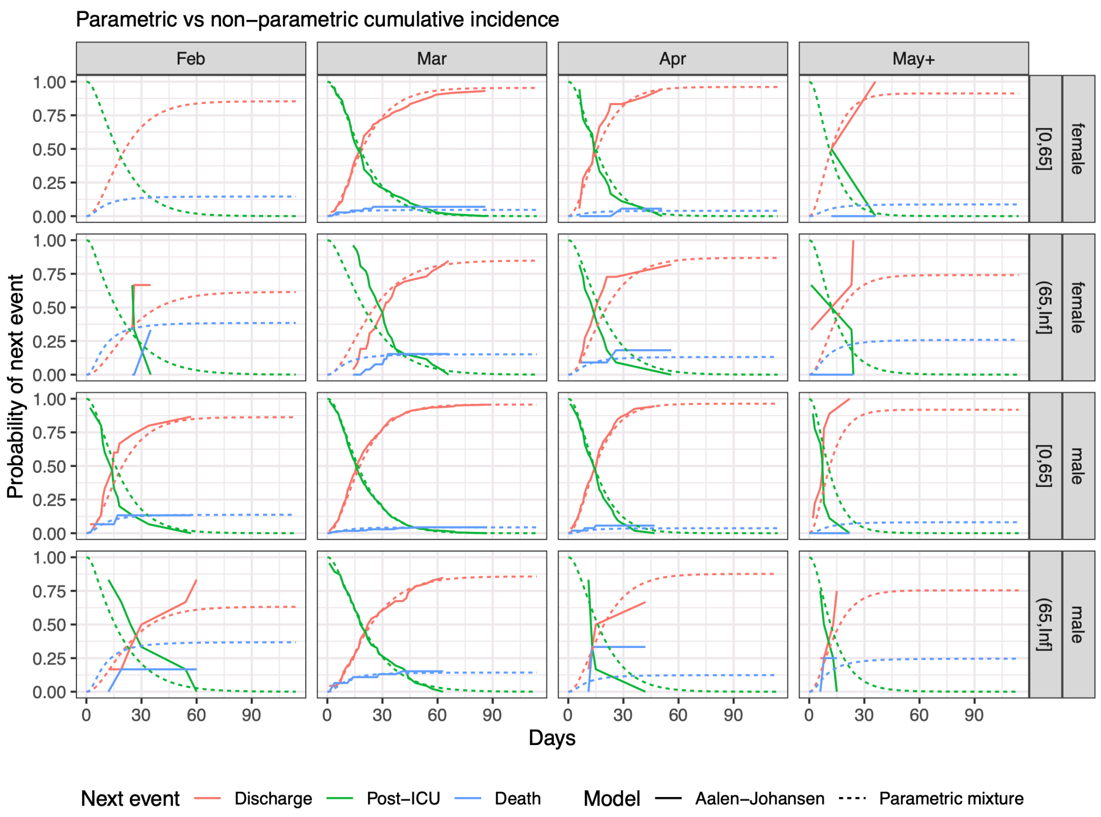

Figure A.30: Cumulative incidence curves for next events from a post-ICU ward, by age, sex, and month of admission.

A.4 Multivariable models - pathway probabilities & times to event

Figure A.31 displays the estimated probabilities of each pathway through hospital, given hospital admission, by sex, age and month of admission, for patients with all other covariates at their baseline values (patients in large hospitals with no comorbidities, who are neither care home residents nor healthcare workers). Note that the probabilities of each pathway, within each sex-age-month stratum, sum to 1. The majority of patients take the pathway Hospital-Discharge, with the probability of this pathway increasing over time, decreasing with age and larger for women than for men.

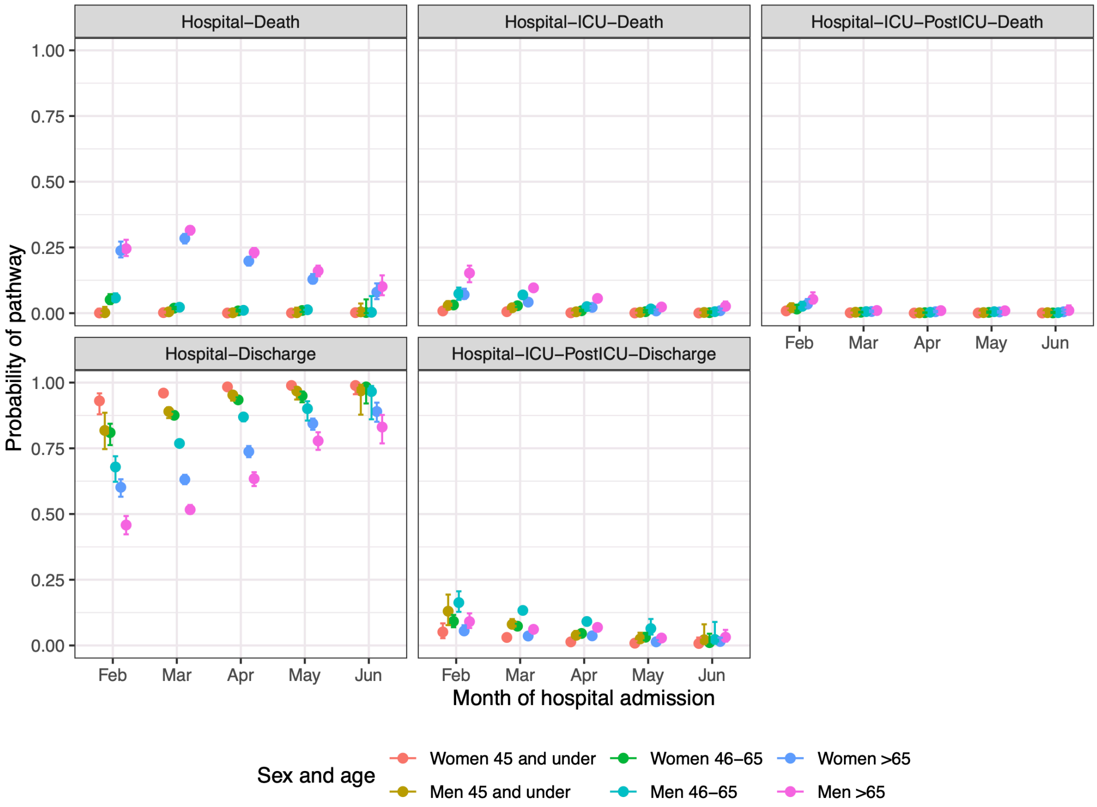

Figure A.31: Estimated probabilities of each pathway through hospital, by sex, age and month of admission.

The distribution of lengths of stay in hospital, by pathway through hospital, are summarised in Figures A.32 (mean time to final event) and A.33 (median and quantiles of distribution of time to final event). Both the mean and median lengths of stay become shorter with calendar month of admission, for both survivors and non-survivors, but this may also reflect the fact that there is less dispersion in the distribution (less heterogeneity in the population) over time. This reduction in heterogeneity over calendar time is seen also in the summaries of the distribution of lengths of stay by final event, averaged over pathway through hospital, in Figures A.34 and A.35.

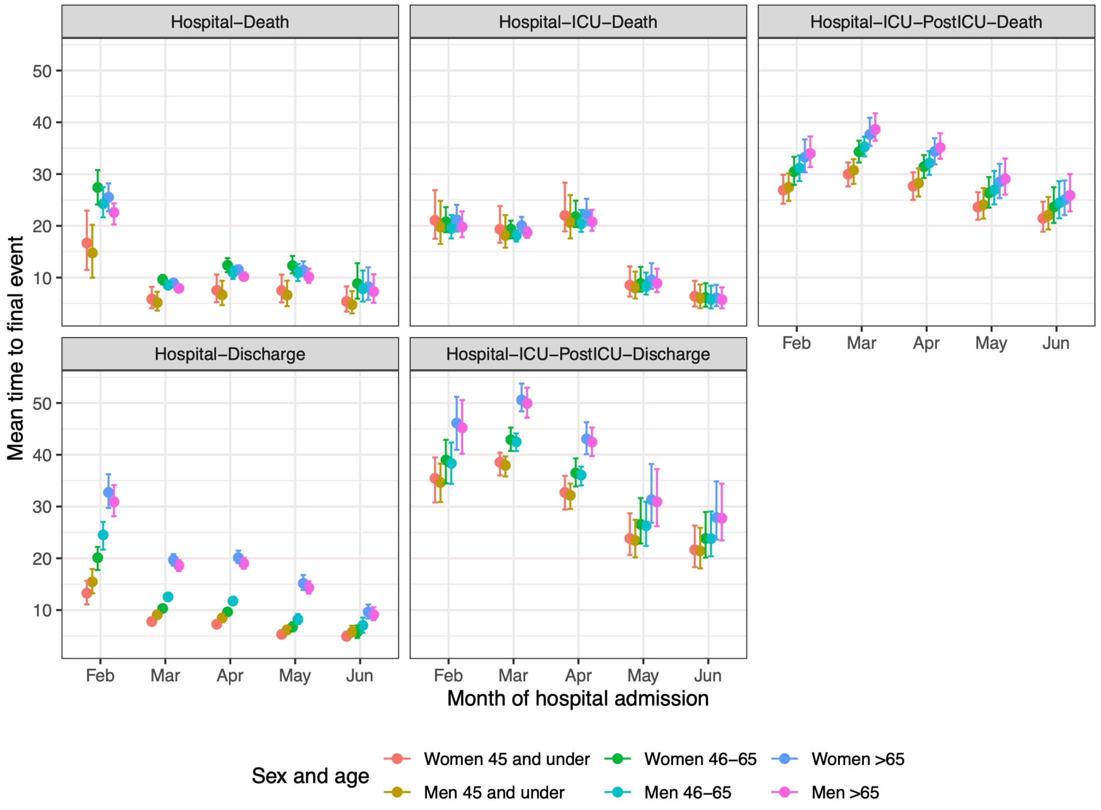

Figure A.32: Estimated mean (95% CI of mean) length of stay in hospital, by pathway through hospital, sex, age and month of admission.

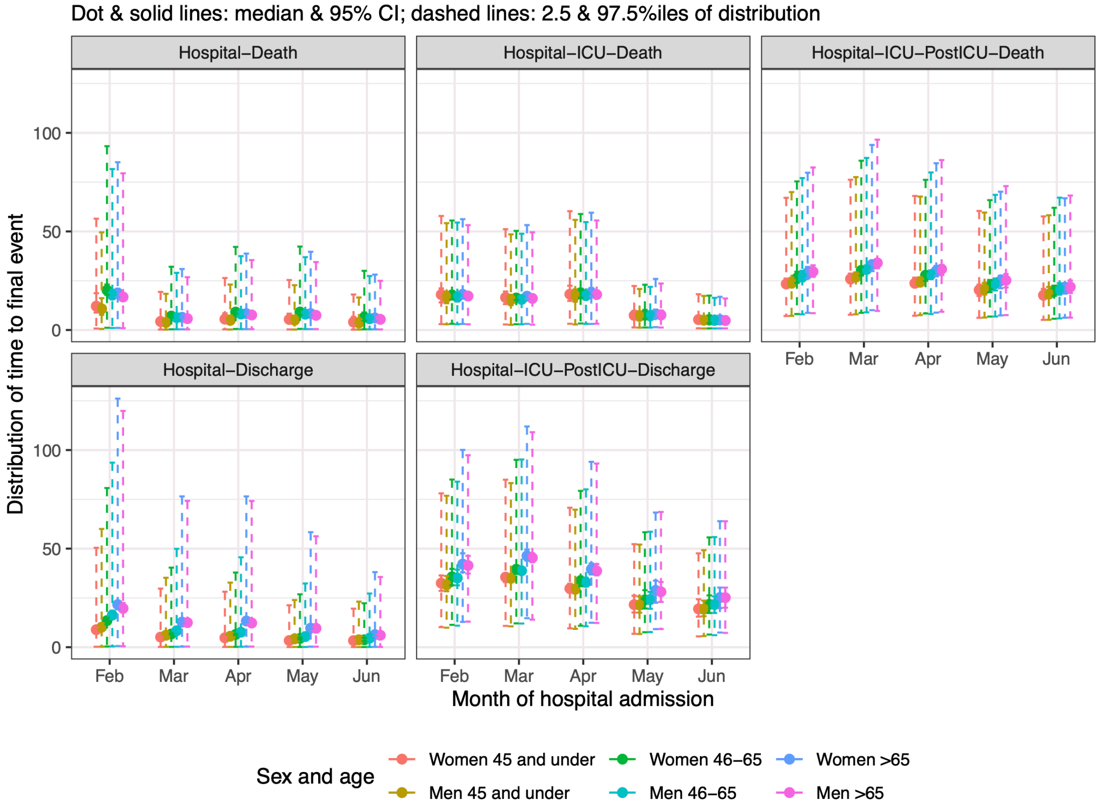

Figure A.33: Estimated median (95% CI of median; 2.5- and 97.5%-ile of distribution of) length of stay in hospital, by pathway through hospital, sex, age and month of admission.

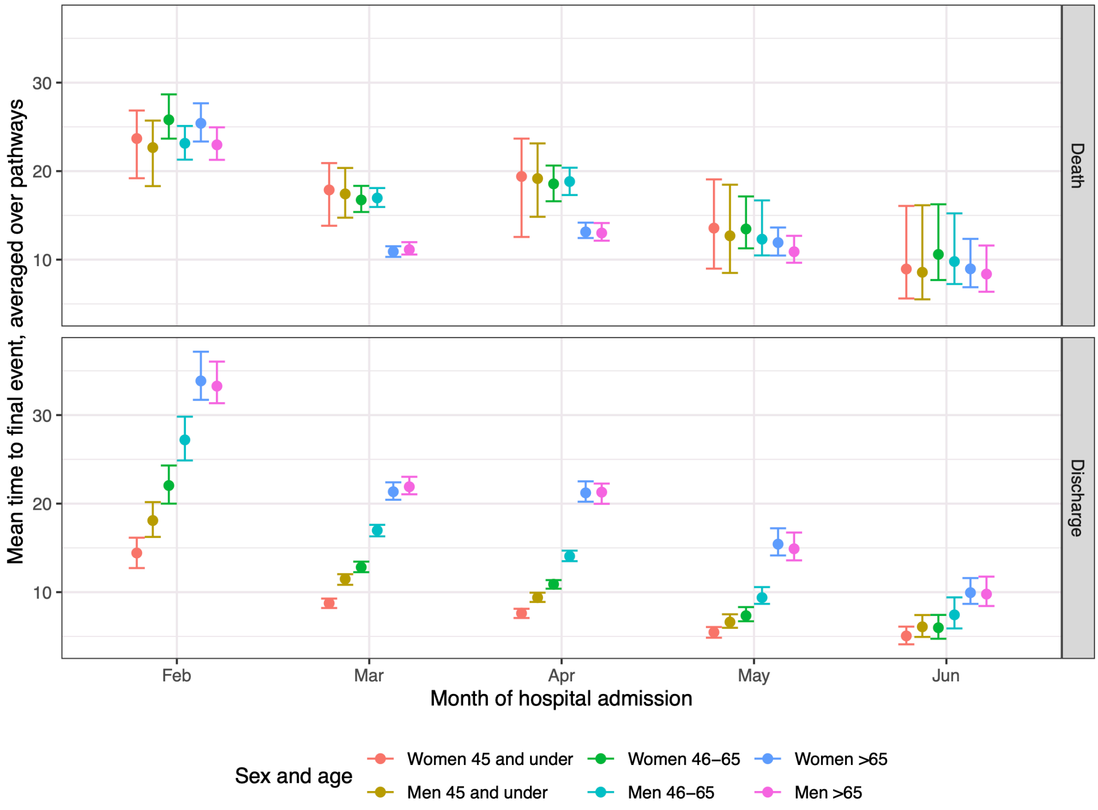

Figure A.34: Estimated mean (95% CI of mean) length of stay in hospital, by final event (averaged over pathway through hospital), sex, age and month of admission.

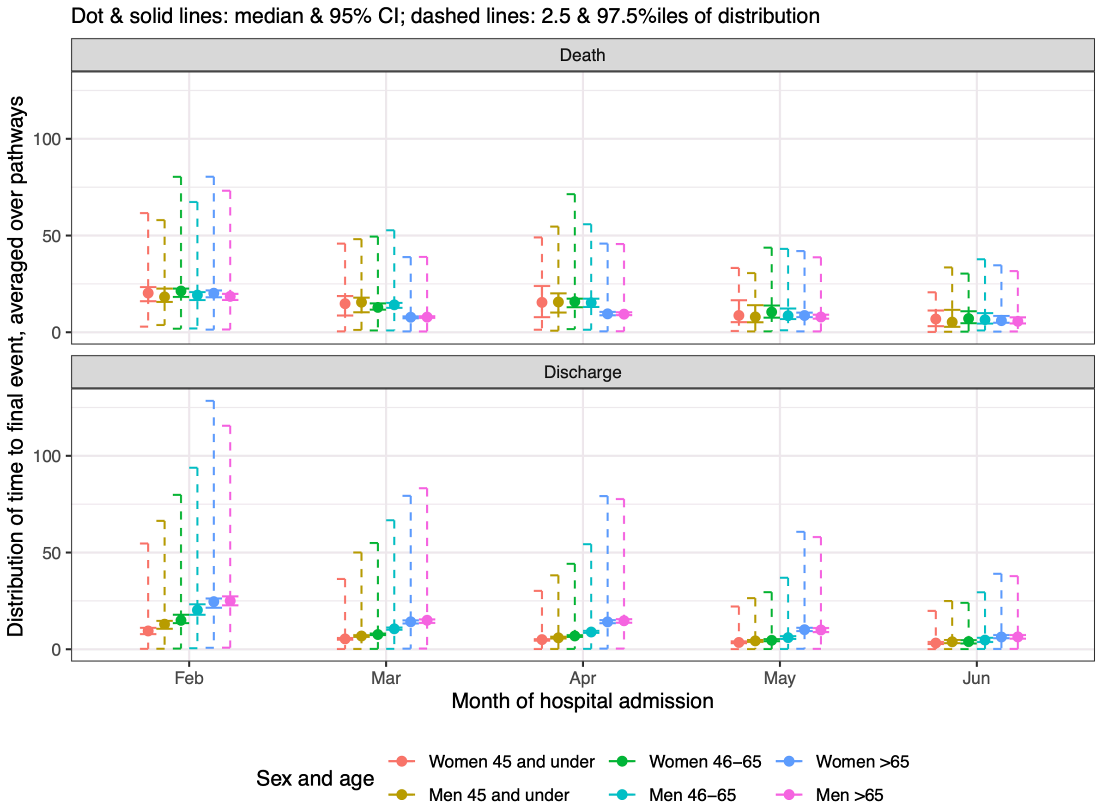

Figure A.35: Estimated median (95% CI of median; 2.5- and 97.5%-ile of distribution of) length of stay in hospital, by final event (averaged over pathway through hospital), sex, age and month of admission.
